# ADAR1-dependent miR-3144-3p editing simultaneously induces MSI2 expression and suppresses SLC38A4 expression in liver cancer

**DOI:** 10.1038/s12276-022-00916-8

**Published:** 2023-01-04

**Authors:** Hyung Seok Kim, Min Jeong Na, Keun Hong Son, Hee Doo Yang, Sang Yean Kim, Eunbi Shin, Jin Woong Ha, Soyoung Jeon, Keunsoo Kang, Kiho Moon, Won Sang Park, Suk Woo Nam

**Affiliations:** 1 Department of Pathology, College of Medicine, The Catholic University of Korea, 222 Banpo-daero, Seocho-gu, Seoul, 06591 Republic of Korea; 2 Functional RNomics Research Center, The Catholic University of Korea, 222 Banpo-daero, Seocho-gu, Seoul, 06591 Republic of Korea; 3 Department of Biomedicine & Health Sciences, Graduate School, The Catholic University of Korea, Seoul, 06591 Korea; 4 Department of Microbiology, Dankook University, Cheonan, 31116 Republic of Korea; 5 NEORNAT Inc., Rm. #5104 Bldg. A, Omnibus Park, 222 Banpo-daero, Seocho-gu, Seoul, 06591 Republic of Korea

**Keywords:** Oncogenes, Liver cancer

## Abstract

Aberrant adenosine-to-inosine (A-to-I) RNA editing, catalyzed by adenosine deaminase acting on double-stranded RNA (ADAR), has been implicated in various cancers, but the mechanisms by which microRNA (miRNA) editing contributes to cancer development are largely unknown. Our multistage hepatocellular carcinogenesis transcriptome data analyses, together with publicly available data, indicated that *ADAR1* was the most profoundly dysregulated gene among RNA-editing enzyme family members in liver cancer. Targeted inactivation of *ADAR1* inhibited the in vitro tumorigenesis of liver cancer cells. An integrative computational analyses of RNA-edited hotspots and the known editing frequency of miRNAs suggested that the miRNA miR-3144-3p was edited by ADAR1 during liver cancer progression. Specifically, ADAR1 promoted A-to-I editing of canonical miR-3144-3p to replace the adenosine at Position 3 in the seed region with a guanine (ED_miR-3144-3p(3_A < G)) in liver cancer cells. We then demonstrated that *Musashi RNA-binding protein 2* (*MSI2*) was a specific target of miR-3144-3p and that MSI2 overexpression was due to excessive ADAR1-dependent over-editing of canonical miR-3144-3p in liver cancer. In addition, target prediction analyses and validation experiments identified *solute carrier family 38 member 4* (*SLC38A4*) as a specific gene target of ED_miR-3144-3p(3_A < G). The ectopic expression of both ADAR1 and the ED_miR-3144-3p(3_A < G) mimic enhanced mitotic activities, and ADAR1 suppressed SLC38A4 expression in liver cancer cells. Treatments with mouse-specific ADAR1-, MSI2-siRNA-, or SLC38A4-expressing plasmids suppressed tumorigenesis and tumor growth in a mouse model of spontaneous liver cancer. Our findings suggest that the aberrant regulation of ADAR1 augments oncogenic MSI2 effects by excessively editing canonical miR-3144-3p and that the resultant ED_miR-3144-3p(3_A < G) simultaneously suppresses tumor suppressor SLC38A4 expression, contributing to hepatocellular carcinogenesis.

## Cancer: Modified microRNA associated with liver tumor growth

Changes made to a small regulatory RNA molecule by RNA-editing enzymes help to promote the growth of liver tumors. Suk Woo Nam from the Catholic University of Korea in Seoul, South Korea, and colleagues showed that in liver cancer cells a microRNA called miR-3144-3p frequently undergoes a common RNA modification that alters its recognition of two target genes, resulting in elevated expression of one and diminished expression of the other. Silencing activity of the former gene or boosting activity of the latter both helped suppress tumor incidence and proliferation in a mouse model of liver cancer. Drugs that target these genes or the upstream pathway mediated by a family of RNA-editing enzymes causing excessive changes to miR-3144-3p could therefore help treat liver cancer.

## Introduction

In cell biology, the epitranscriptome comprises post-transcriptional RNA modifications, including methylation, splicing, and RNA editing, that lead to various functional changes in the transcriptome^1^. Among these alterations, RNA editing is a widespread co or post-transcriptional modification that introduces changes to RNA sequences encoded by the genome^1,2^. The best-characterized examples of RNA editing in mammals involve the conversion of cytosine to uracil (C-to-U) and adenosine to inosine (A-to-I)^3^. In humans, the most frequent RNA-editing mutation is the A-to-I conversion, which is catalyzed by a double-stranded RNA (dsRNA)-specific adenosine deaminase acting on RNA (ADAR) family of proteins, composed of ADAR1, ADAR2, and ADAR3, all of which carry dsRNA-binding domains^4^.

In general, as a result of RNA editing, inosine bases are interpreted by the cellular machinery as guanosine and are base pared with cytosine such that the of A-to-I substitution is similar to the A-to-G substitution. These changes can lead to specific amino acid substitutions, alternative splicing, microRNA (miRNA)-mediated gene silencing, and/or changes in transcript localization and stability. Aberrant RNA editing is an underexplored mechanism that reproducibly alters protein and regulatory RNA sequences that drives carcinogenesis and therefore is a potential therapeutic target^5^. Indeed, A-to-I editing and the enzymes mediating this modification have been shown to be significantly altered in cancer. In most tumor types, the RNA-editing activity is higher than that in matched normal tissues, but in certain cancers, RNA has been reported to be underedited^6^. The vast majority of A-to-I RNA-editing events occur in noncoding regions, such as untranslated regions (UTRs) and introns, and in long noncoding RNAs, and miRNAs. In addition, systematic characterization of A-to-I editing hotspots in miRNAs across many types of human cancer has suggested the importance of miRNA editing in gene regulation and the edited product as a potential biomarker for cancer prognosis and therapy^7^.

The editing of RNA with miRNAs shows the potential to regulate the processing of precursor miRNAs into mature miRNAs^8^. Additionally, A-to-I editing of primary miRNA by ADARs interferes with miRNA biogenesis and thus alters miRNA homeostasis. Moreover, because miRNA regulation requires perfect base pairing within a seed region (2–8 positions) of an miRNA, a single nucleotide change can alter miRNA target recognition^9^. Notably, a single nucleotide change can alter the base-pairing properties of a miRNA, and therefore, editing within a seed sequence of a miRNA may alter the recognition of a target gene by eliminating the original target or by acquiring new targets. Intriguingly, several miRNA-editing events appear to be critical in cancer. For example, an edited miR-455 mutant has been postulated to suppress tumor growth and metastasis by upregulating tumor suppressor *CPEB1* expression in melanoma^10^. In contrast, a recent study reported that ADAR1-mediated miR-200b overexpression contributes to thyroid cancer^11^. On the other hand, it has been reported that aberrant editing of mRNAs of specific genes, such as *AZIN1*, *FLNB*, and *COPA*, contributes to the development of liver cancer^12^. Nevertheless, ADAR-dependent editing of noncoding RNA remains to be studied.

In this study, we investigated the oncogenic function of ADAR1 by promoting miR-3144-3p editing to simultaneously induce *Musashi RNA-binding protein 2* (*MSI2*) and suppress *solute carrier family 38 member 4* (*SLC38A4*) in human liver cancer. We explored the transcriptome and small-RNA-sequencing data of human multistage liver disease, including chronic hepatitis, cirrhosis, dysplastic nodules, and liver cancers. From these analyses, we suggest that, among the RNA-editing enzyme family members, ADAR1 was particularly overexpressed and thus identified miR-3144-3p as an ADAR1 editing target in liver cancer. We then showed that ADAR1 overexpression and resulting miR-3144-3p editing augmented the aggressiveness of liver cancer cells through their effects on tumor cell growth, proliferation, invasion, and migration and in vivo tumor growth. Notably, we demonstrated that ADAR1 promoted excessive miR-3144-3p editing, especially at adenine Position 3 in the sequence of the canonical miR-3144-3p seed region, to produce the guanine-carrying miR-3144-3p (ED_miR-3144(3_A < G)) mutant in liver cancer cells. This critical ADAR1-dependent change in the canonical miR-3144-3p seed region attenuated the negative regulation of canonical miR-3144-3p activity on *MSI2* mRNA and simultaneously created a novel edited mutant of miRNA, ED_miR-3144(3_A < G), which inhibited the mRNA translation of the tumor suppressor *SLC38A4* in liver cancer cells. Thus, in this study, we identified the pathogenic activity of ADAR1-mediated miRNA editing that suppressed canonical miRNA activity and created an edited miRNA mutant that potentiated the malignant transformation and growth of hepatocytes.

## Materials and methods

### Tissue samples

A total of 36 matched pairs of liver cancer tissues and their corresponding noncancerous liver tissues were obtained from AJOU University Hospital and Keimyung University Hospital, a member of the National Biobank of Korea. Written informed consent was obtained from each subject following guidance of the Declaration of Helsinki, and the study was approved by the Institutional Review of Board (IRB) of the Songeui Campus, College of Medicine, the Catholic University of Korea (IRB approval numbers MC18TESI0075 and MC19TESI0016).

### Cell culture

Human liver cancer cells (Hep3B, HepG2, Huh7, PLC/PRF/5, SK-HEP-1, SNU-182, SNU-354, SNU-368, SNU-387, SNU-423, SNU-449, and SNU-475 cells) were obtained from KCLB (Korean Cell Line Bank, Seoul, Korea). The normal liver cell line MIHA was kindly provided by Dr. Roy-Chowdhury (Albert Einstein College of Medicine, Bronx, NY). All of the cell lines were maintained in RPMI-1640 or DMEM medium with 10% fetal bovine serum and 100 units/ml penicillin/streptomycin (GenDepot, Katy, TX). All cells were cultured at 37 °C in a humidified incubator with 5% CO_2_.

### Transfection and treatment

Small interfering RNAs (siRNAs) were synthesized by Genolution (Seoul, Korea) or purchased from BIONEER (Daejeon, Korea). The sequences of the siRNAs, miRNA mimics, and antisense miRNAs are listed in the Supplementary Table 5. The human ADAR1-p110, MSI2, and SLC38A4 expression plasmids and the subcloning gene ORF sequence in the pcDNA3.1 + /C-(K)-DYK plasmid were purchased from GenScript^TM^ (Piscataway, NJ, USA). Transfection was carried out using Lipofectamine RNAiMAX or Lipofectamine 2000 reagent (Invitrogen, Carlsbad, CA, USA) according to the relevant manufacturer’s instructions.

### RNA, DNA extraction, RT-PCR, and qRT-PCR

Total RNA from frozen tissues and cells were isolated using TRIzol reagent (Invitrogen, Carlsbad, CA, USA). One microgram of total RNA was reverse transcribed into cDNA using a Tetro cDNA Synthesis kit (Bioline, London, UK) according to the manufacturer’s instructions. Quantitative real-time PCR (qRT‒PCR) was performed with a SensiFAST SYBR No-ROX kit (Bioline, London, UK) and monitored in real time with an iQ™-5 system (Bio–Rad, Hercules, CA, USA). The average threshold cycle (Ct) value obtained through triplicate assays was used for further calculations. Normalized gene expression was determined using the relative quantification method. The results are expressed as the mean value of triplicate experiments. Genomic DNA from tissue and cells was isolated using DNAzol reagent (Invitrogen, Carlsbad, CA, USA) according to the manufacturer’s instructions. qRT-PCR was performed as described above, and glyceraldehyde-3-phosphate dehydrogenase (GAPDH) was used as the endogenous loading control. The sequences of the primers used for qRT‒PCR are listed in the Supplementary Table 6.

### FLAG immunoprecipitation

Cells were transfected with a pcDNA3.1_ADAR1-p110 or pcDNA3.1_MSI2 plasmid, which encodes a FLAG-tag. Forty-eight hours after transfection, the cells were washed with phosphate-buffered saline (PBS) and lysed at 4 °C in PBS, pH 7.2, supplemented with 1.0% NP-40, 0.5% sodium deoxycholate, 0.1% SDS, 10 mM NaF, 1.0 mM NaVO4, and a 1.0% protease inhibitor cocktail (Sigma). The FLAG-tag was immunoprecipitated with anti-FLAG DynaBeads (Invitrogen, Carlsbad, CA) during an overnight incubation. Immunoprecipitated proteins were eluted using a 3× FLAG peptide (Sigma) and analyzed by western blot probing with an anti-FLAG antibody (Cell Signaling Technology). For primary miR-3144 pull-down analysis and qRT-PCR, RNA was isolated and reverse transcribed using a miScript II RT kit (Qiagen).

### Western blot analysis

Cells were lysed using lysis buffer (50 mM HEPES, 5 mM EDTA, 50 mM NaCl, 1% Triton X100, 50 mM NaF, 10 mM Na2P4O7, 1 mM Na3VO4, 5 μg/mL aprotinin, 5 μg/mL leupeptin, 1 mM PMSF, and a protease inhibitor cocktail). Lysates containing equal amounts of proteins were separated by SDS-PAGE and transferred onto a polyvinylidene difluoride membrane (Bio–Rad, Hercules, CA, USA). The membrane was blocked with a 5% skim milk solution and incubated with the following antibodies: anti-ADAR1, anti-CTNNB1, anti-GAPDH, anti-MSI2, anti-MET, anti-SLC38A4 (Santa Cruz Biotechnology, Dallas, TX, USA), and anti-FLAG-Tag (Cell Signaling Technology, Danvers, MA, USA). An Immobilon™ Western blot detection system (Millipore) was used to detect bound antibodies. The intensities of the western blot bands were quantified using LAS-4000 (Fuji Photo Film Co., Tokyo, Japan).

### Cell growth assay

Cells were seeded in a six-well plate and transfected. After transfection, the cells were incubated with a 0.5 mg/ml MTT [3-(4,5-dimethylthiazol-2-yl)-2,5-diphenyltetrazolium bromide] solution (Sigma) for 1 h. The dark blue formazan formed in viable cells was dissolved in dimethyl sulfoxide (DMSO; Sigma), and the absorbance was measured using a VICTOR3 Multilabel plate reader (PerkinElmer, Waltham, MA).

### Bromodeoxyuridine (BrdU) incorporation assay

Cells were seeded in a 24-well plate to 40–50% confluency. The assay was performed with a bromodeoxyuridine (BrdU) cell proliferation assay kit (Millipore) following the manufacturer’s protocol every 24 h.

### Clonogenic assay

Cells were transfected with miRNA mimics or siRNA in 60 mm^2^ cell culture plates. After transfection for 24 h, the cells were reseeded in six-well plates and incubated for 2 weeks. Next, the cells were washed with PBS and fixed with 1% paraformaldehyde for 30 min at room temperature. The fixed cells were stained with 0.5% crystal violet for 1 hr at room temperature. Colonies were counted using a clono-counter program.

### Apoptosis assay

To measure the apoptosis rate, an Annexin V-FITC Apoptosis Detection Kit I (BD Biosciences, San Jose, CA) was used. After transfection, liver cancer cells were washed with cold phosphate-buffered saline (PBS) and resuspended in 1× binding buffer. Then, 1 × 10^5^ cells were transferred to a 5 ml culture tube and mixed with 5 μl of annexin V-FITC and 10 μl of a propidium iodide solution. After 20 min at room temperature in the dark, 400 μl of 1× binding buffer was added to each tube, and the number of apoptotic cells in each fraction was measured with a FACSCalibur flow cytometer (BD Biosciences).

### Cell cycle analysis

Liver cancer cells were transfected with miRNA mimics or siRNA in 60 mm^2^ dishes. After 48 h of incubation, the cells were harvested with trypsin, washed with cold PBS, fixed in 70% ethanol, resuspended in 200 μl of PBS supplemented with 1 mg/ml RNase, and incubated in the dark for 30 min at 37 °C. Nuclei were stained with 50 μg/mL propidium iodide (BD Biosciences). The percentage of stained cells in each fraction was determined using Cell-Quest FACS analysis software (BD Biosciences).

### Migration and invasion assays

For in vitro cell migration and invasion assays a modified Boyden chamber was used to determine cell motility. For the invasion assay, Matrigel (BD Biosciences) was diluted with coating buffer to a final concentration of 0.3 mg/ml. One hundred microliter aliquots of this Matrigel concentrate were used to coat the upper surface of Transwell cell culture inserts. After incubation for 2 h at 37 °C, the inserts were seeded with cells. After preparation the cells were plated on the top surfaces of the Transwell inserts, the inserts were placed in a 24-well plate. The lower wells contained a 2% FBS chemoattractant. The plate was incubated overnight and stained using a Diff-Quik staining kit (Sysmex, Kobe, Japan). The cells were imaged using an Axiovert 200 inverted microscope (Zeiss, Oberkochen, Germany) at ×200 magnification, and the number of cells in three random image fields were counted.

### Wound-healing assay

Cells were transfected and incubated for 24 h in 60 mm^2^ cell culture plates. Then, the cells were trypsinized, and 1 × 10^6^ cells were seeded per well in a six-well cell culture plate. After overnight incubation, the cell monolayers were scraped with a sterile micropipette tip. Initial gap widths 0 h after scratching and residual gap widths 24 h after scratching were photographed with an IX71 photomicrograph (Olympus, Tokyo, Japan).

### Mutagenesis

For mutagenesis of ADAR1-p110, which shows adenosine deaminase activity, a QuickChange kit (Agilent Technologies, Palo Alto, CA, USA) was used according to the manufacturer’s instructions.

### Mouse liver cancer model

The H-*ras* homozygous transgenic mice were kindly provided by Dr. Dae-Yeoul Yu (Laboratory of Human Genomics, Korea Research Institute of Bioscience and Biotechnology, Daejeon, Korea)^13^. Male mice spontaneously developed liver cancer beginning at ~15–18 weeks of age. Mouse livers were harvested at 24 weeks of age and processed for experiments. All animal research procedures were performed in accordance with the Laboratory Animals Welfare Act, the Guide for Care and Use of Laboratory Animals, and the Guidelines and Policies for Rodent Experiment provided by the IACUC (Institutional Animal Care and Use Committee) at the School of Medicine, The Catholic University of Korea (approval number: CUMS-2019-0115-02)

### Mfuzz clustering

The gene expression profiles were log-normalized and clustered using the c-mean algorithm in the Bioconductor Mfuzz package v. 2.30.0 following the author’s instructions^14^.

### Analyses of publicly available genomic data

To investigate the differential gene expression of coding and noncoding RNAs in multistage liver disease, data were obtained from The Cancer Genome Atlas liver cancer project (TCGA_LIHC), the International Cancer Genome Consortium Liver Cancer–RIKEN, JP (ICGC_LIRI) and the Gene Expression Omnibus (GEO) database of the National Center for Biotechnology Information (NCBI) (Accession Numbers: GSE6764, GSE77314, GSE114564, and GSE174608). Level 3 mRNA expression data from TCGA-LIHC HTSeq-FPKM were log2 transformed [log2(fpkm+1)] and used to assess gene expression.

### MicroRNA target prediction

An in silico analysis was performed to predict target candidates of miR-3144-3p and ED_miR-3144(3_A < G) using the TargetScan algorithm (http://www.targetscan.org/). The sequence information of miR-3144-3p was obtained from the miRbase database (http://www.mirbase.org).

### Analysis of miRNA A-to-I editing using Catholic_mLIHC and TCGA_LIHC datasets

All bam files were converted to fastq format using bedtools bamtofastq. To remove adaptors, and low-quality, including reads of inadequate length, from our human multistage liver cancer transcriptome data (Catholic_mLIHC) and TCGA_LIHC data, Cutadapt was used with the “-a adaptor_sequence --quality-base 33 -m 15 -M 28 -f fastq -O 3 -q 20” options. Next, Bowtie was used to align the filtered reads to the human genome (hg19), and the “-n 1 –e 50 –a –m --best --strata --trim3 2” option was chosen for performing the best alignment with one mismatch per read and no cross mapping. In the miRNA-editing-profiling stage, all procedures were performed using the scripts reported in Bazak, L et al.^15^. Briefly, first, binomial test was performed, and only candidates with a Bonferroni-corrected *p*-value of 0.1 or less were selected. Second, all SNPs in sites other than a pre-miRNA, including those in mitochondria, were removed. Finally, to exclude all SNPs that had been previously reported, all overlapping mutation information recorded in the dbSNP and gnomAD databases was removed. To obtain more meaningful results, miRNAs with an average editing rate of 5% or more and a TPM expression value of 1 or more were selected from the Catholic_mLIHC dataset, and miRNAs with an editing rate of 5% or more in at least 10 samples were selected from the TCGA_LIHC dataset.

### In vivo tumorigenesis study

Ras-Tg mice were intravenously injected with Invivofectamine 3.0 (Invitrogen, Carlsbad, CA, USA) supplemented with 0.25 mg/kg Adar1 and Msi2 siRNAs as previously described^16^. the Ras-Tg mice were also intravenously injected with 50 µg of a pcDNA3.1_Slc38a4 plasmid using Turbofect in vivo Transfection Reagent (Invitrogen, Carlsbad, CA, USA). The ultrasonography images were taken at 17, 19, 21, and 23 weeks of age with an ultrasound machine (Philips, Amsterdam, Nederland) by the same medical imaging expert each time.

### Statistical analyses

Survival curves were plotted using the Kaplan‒Meier product limit method, and significant differences between survival curves were determined via log-rank test. All experiments were performed at least three times, and all samples were analyzed in triplicate. The statistical significance of a difference between experimental groups was assessed by paired or unpaired Student’s *t*-tests using GraphPad 7.0 software (GraphPad Software Inc.). Statistical significance was determined to be a *p*-value <0.05. Chi-square test (two-sided) was performed to determine associations between parameters. Receiver operating characteristic (ROC) curves for each candidate marker were analyzed to calculate sensitivity, specificity, and areas under the curve (AUCs) at a 95% confidence interval.

## Results

### Identification of ADAR1 as a potential RNA-editing factor in liver cancer

**Fig. 1:**
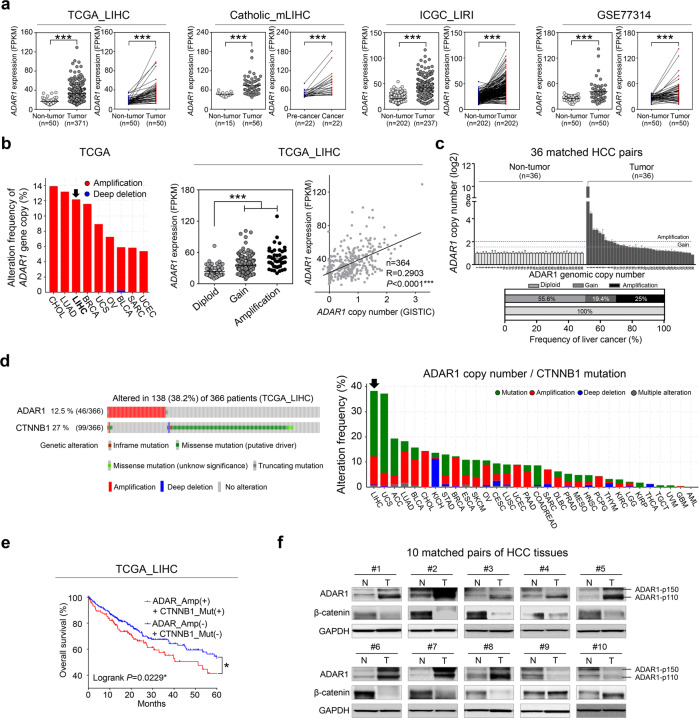
*ADAR1* is overexpressed via genomic amplification, and the mutations to *ADAR1* and *CTNNB1* are mutually exclusive in liver cancer. **a** Differential gene expression of *ADAR1* in sets of RNA sequencing data from liver cancer patients compared with that in healthy normal patients (nontumor) and matched pairs of liver cancer tissues. **b** *ADAR1* genomic alteration in various cancers as determined with cBioPortal (left). *ADAR1* expression change due to genomic alteration, as determined with the TCGA_LIHC dataset (middle). The correlation of *ADAR1* gene expression with its genomic copy number, as determined with the TCGA_LIHC dataset (right). **c** qRT‒PCR analysis of *ADAR1* gene copy number in matched human liver cancer tissues. **d** OncoPrint showing genomic alterations in *ADAR1* and *CTNNB1* in liver cancer patients, as determined with the TCGA_LIHC dataset. Mutual exclusivity between *ADAR1* amplification and *CTNNB1* mutation was analyzed (left). A systemic analysis of the merged alteration frequencies of *ADAR1* and *CTNNB1* in TCGA dataset (right). **e** Kaplan‒Meier survival curves with *ADAR1* gene amplification (Amp) and *CTNNB1* mutation (Mut) in liver cancer patients. **f** Western blot analysis of ADAR1 and β-catenin in 10 selected matched pairs of tumor (T) and adjacent nontumor (N) tissues from liver cancer patients. All data are shown as the mean ± SEM; *P < 0.05, **P < 0.01, ***P < 0.001 by unpaired Student’s *t*-test.

A previous study reported that aberrant RNA editing, especially A-to-I editing, is mediated by ADARs in human liver cancer, but the precise mechanism by which this editing contributes to liver cancer had not yet been identified^17^. Considering this previous report, we recapitulated the differential expression of RNA-editing gene families in publicly available datasets (the TCGA_LIHC, ICGC_LIRI, and GSE77314 dataset) and our multistage liver cancer (Catholic_mLIHC; GSE114564) transcriptome data. We found that *ADAR1* was significantly overexpressed in liver cancer patients (≥±1.5-fold, *P* < 0.05), and *APOBEC3B* was also significantly upregulated in the same datasets (Fig. 1a and Supplementary Table 1). However, a comparative gene expression analyses of *ADAR1* and *APOBEC3B* using multistage liver cancer datasets showed liver-cancer-specific expression of *ADAR1* (Supplementary Fig. 1a, b), and a ROC analysis showed that *ADAR1* expression was more specific than *APOBEC3B* to liver cancer (Supplementary Fig. 1c, d). Next, we investigated genetic alterations in *ADAR1* recorded in TCGA datasets. Genomic amplification at the *ADAR1* locus was found to be frequent (12.5%), and this genetic alteration was significantly correlated with *ADAR1* mRNA expression in liver cancer (Fig. 1b). A PCR-based copy number analysis of 36 selected matched pairs of human liver cancer tissues revealed alterations in 44% of the *ADAR1* gene copies in liver cancer (gain (*n* = 7) and amplification (*n* = 9)) (Fig. 1c). Notably, *ADAR1* gene amplification and mutation of the *CTNNB1* gene, encoding β-catenin protein B, were mutually exclusive in liver cancer in the TCGA_LIHC dataset, this mutation was found to be most prevalent among all the mutations (38.2%, *n* = 138) in the 32 cancer types investigated in TCGA Pan-Cancer Atlas, and this mutually exclusive tendency was found only in liver cancer (Fig. 1d and Supplementary Table 2). In addition, in the Kaplan‒Meier survival analysis of the TCGA_LIHC data derived from liver cancer patients, alterations in *ADAR1* and *CTNNB1* were associated with a poorer prognosis than that obtained for patients without these alterations (Fig. 1e). We then performed both Western blot and qRT-PCR analyses and confirmed that 9 of 12 tested liver cancer cell lines exhibited ADAR1 overexpression compared with its expression in MIHA cells, an immortalized untransformed hepatocyte cell line (Supplementary Fig. 2a, b). By performing Western blot analyses, we also found that ADAR1 was highly expressed in liver cancer, but β-catenin was not expressed in 10 liver cancer tissues (Fig. 1f).

### The tumorigenic potential of ADAR1 and identification of miRNA editing in liver cancer

**Fig. 2:**
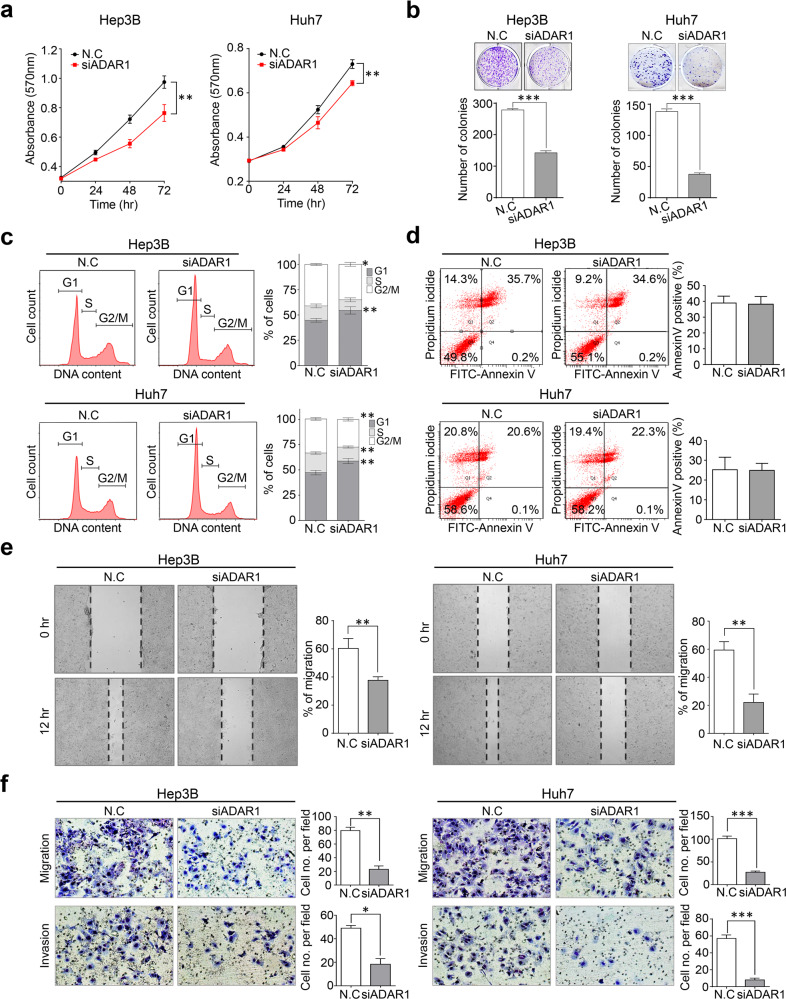
Targeted inactivation of ADAR1 suppresses the tumorigenic potential of liver cells. **a** Cell growth was measured by MTT assay. **b** Anchorage-independent growth was determined by clonogenic assay. **c** Flow cytometry with propidium iodide (PI)-positive cells after treatment with control siRNA (N.C.) and ADAR1-specific siRNA (siADAR1), respectively (left). The PI-stained cell number to total cell number ratios are presented with a bar graph (right). **d** The apoptosis rates of the cancer cells stained with annexin V-FITC and propidium iodide were evaluated after ADAR1 knockdown via flow cytometry (left). The annexin V-FITC-stained cell number to total cell number ratios are presented with a bar graph (right). **e** Scratch wound-healing assay (left), and the ratios of the remaining gap size to the original gap size are represented with a bar graph (right). **f** Transwell migration and invasion assays. All data are shown as the mean ± SEM. *P < 0.05, **P < 0.01, ***P < 0.001 by unpaired Student’s *t*-test.

To identify the functional roles of ADAR1 in liver cancer, we performed in vitro tumorigenesis assays. We found that ADAR1 knockdown significantly suppressed the growth and proliferation of Hep3B and Huh7 cells (Fig. 2a, b). In addition, flow cytometry analyses indicated that ADAR1 knockdown significantly increased the G1 arrest rate but exerted no effect on death processes in these cells (Fig. 2c, d). Moreover, ADAR1 knockdown was correlated with antitumorigenic effects in scratch wound healing and in vitro cell motility, as indicated by cell invasion assay (Fig. 2e, f).

Considering these results, we searched for miRNAs that are targets of the edited ADAR1 mutant in liver cancer. Among potentially edited miRNAs, only miRNAs with an editing site included in the miRNA seed region were selected from the small-RNA-sequencing data obtained from both the Catholic_mLIHC and TCGA_LIHC datasets. Then, to identify the miRNAs carrying high-confidence RNA-editing hotspots, miRNAs with A-to-I editing rates of 5% or more were selected. Finally, in the Catholic_mLIHC datasets, miRNAs for which the editing frequency increased as liver cancer progressed were selected, and in the TCGA_LIHC datasets, miRNAs for which the editing frequency was increased in liver cancer compared with that in normal tissue were selected. In this way, four miRNAs—miR-1304-3p, miR-3144-3p, miR-499a-3p, and miR-589-3p—commonly to both datasets were included in the final analyses (Supplementary Fig. 3a). Of these targets, miR-3144-3p was found to be associated with A-to-I editing in the seed region, and the editing frequency was confirmed to be simultaneously increased as the liver cancer progressed through stages and to significantly increased compared with that in normal liver tissue (Supplementary Fig. 3b, c). Notably, the editing frequency rate and miR-3144-3p level increased as the *ADAR1* gene copy number increased (Supplementary Fig. 3d).

**Fig. 3:**
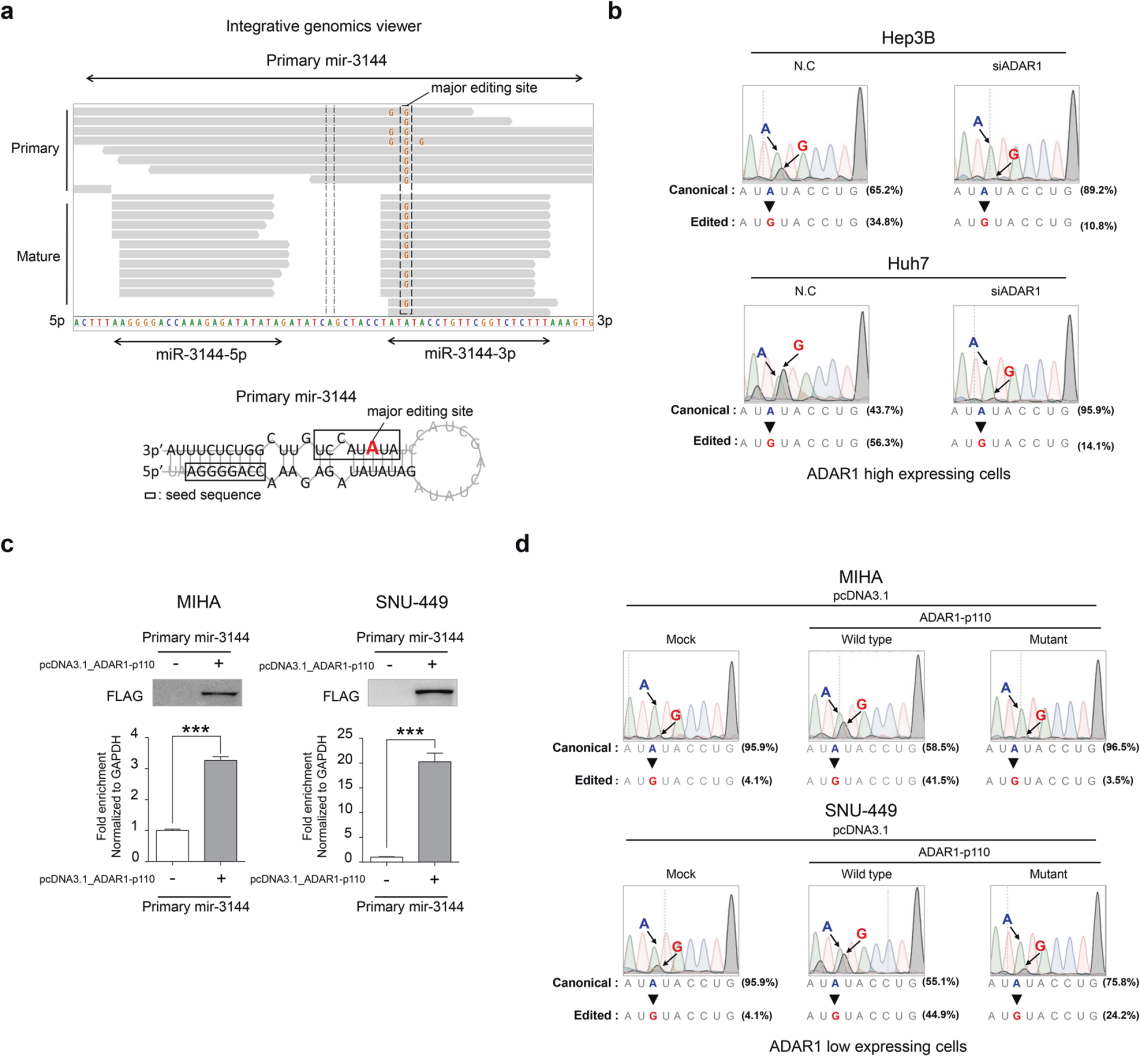
ADAR1-dependent A-to-I editing of canonical miR-3144-3p in liver cancer. **a** Representative integrative genomics viewer (IGV) image of A-to-I edited sites in the primary mir-3144 (upper) sequence and seed region of mature miR-3144 (lower) in liver cancer patients. Red indicates a major editing site in precursor mir-3144. **b** Direct sequencing analyses after ADAR1 knockdown in liver cancer cell lines with high ADAR1 expression. **c** RNA immunoprecipitation assay with ADAR1-overexpressing cells. The fold enrichment of primary mir-3144 was measured by qRT-PCR and normalized to the level of GAPDH. **d** Direct sequencing analyses of miR-3144-3p in wild-type or mutant ADAR1-overexpressing cells. All data are shown as the mean ± SEM; *P < 0.05, ***P < 0.001 by unpaired Student’s *t*-test.

Next, to confirm the editing of miR-3144-3p in liver cancer patients, we searched editing sites of both full-length primary miRNA 3144 (mir-3144) and sequences in the mature form of miR-3144 in Catholic_mLIHC datasets. Notably, among the entire mir-3144 sequence, A-to-I (G) editing sites were identified only in the mature miR-3144-3p sequence, particularly in the seed region, and we confirmed that the frequency of these editing events increased as liver cancer progressed through stages (Fig. 3a; Supplementary Fig. 4a). Then, after ADAR1 knockdown in the Hep3B and Huh7 liver cancer cell lines, direct sequencing of the target site of the seed region revealed an increase in the canonical form, that is, with adenine (A), together with a reduction in the edited form, that is, with guanine (G) (Fig. 3b). Next, to investigate whether the seed region mutation in miR-3144-3p is directly catalyzed by ADAR1, we prepared two different plasmids, pcDNA3.1_ADAR1-p110_wild (the active functional form of ADAR1-p110 in liver cancer) and pcDNA3.1_ADAR1-p110_mutant (the form carrying a mutation in the adenine deaminase domain) (Supplementary Fig. 4b). We observed significant enrichment of primary mir-3144 when pcDNA3.1_ADAR1-p110_wild was transfected into the MIHA and SNU-449 cell lines, which show relatively low expression of ADAR1 (Fig. 3c). Direct sequencing of the major editing site in the seed region revealed that introducing enzymatically active ADAR1 (pcDNA3.1_ADAR1-p110_wild) induced the mutation leading to the guanine (G) edited form, whereas the mutant form of ADAR1 (pcDNA3.1_ADAR1-p110_mutant) did not lead to A-to-I (G) editing in the seed region of miR-3144-3p (Fig. 3d).

### The identification of targets of canonical and edited miR-3144-3p in liver cancer

**Fig. 4:**
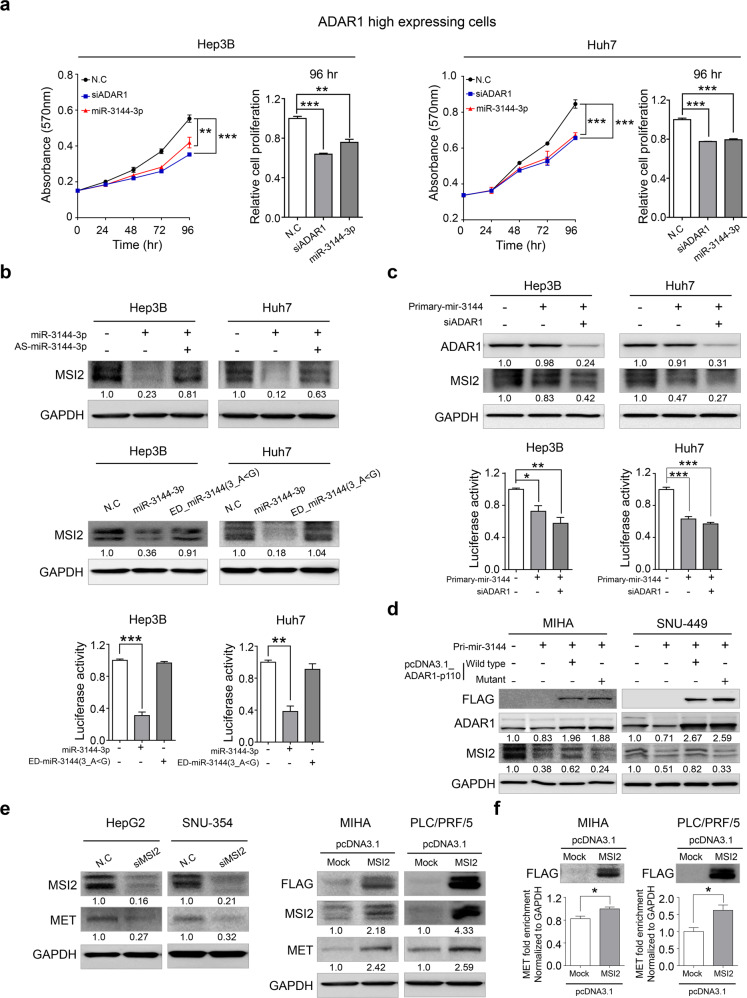
Activation of oncogenic MSI2 by ADAR1-dependent editing of canonical miR-3144-3p in liver cancer. **a** ADAR1 knockdown and miR-3144-3p suppression of liver cancer growth, as determined by using the MTT assay. **b** Antisense miR-3144-3p (upper), but not the ED_miR-3144(3_A < G) mimic, attenuated the suppression of MSI2 mediated by canonical miR-3144-3p in liver cancer cells (middle and lower). **c** Western blot (upper) and luciferase reporter assays (lower) showing MSI2 regulation by ADAR1-dependent primary mir-3144 editing in liver cancer cells. **d** ADAR1-dependent MSI2 expression was measured by immunoblot analysis. **e** Western blotting was performed after MSI2 expression was knocked down (left) or pcDNA3.1-MSI2 was overexpressed (right). **f** the MET transcript was pulled down with pcDNA3.1-MSI2, and the degree of MET abundance was measured by qRT-PCR. All data are shown as the mean ± SEM; *P < 0.05, **P < 0.01, ***P < 0.001 by unpaired Student’s *t*-test.

In liver cancer cells highly expressing ADAR1, transfection with a miR-3144-3p mimic showed a significant inhibitory effect on cell growth similar to that of ADAR1 knockdown, implying attenuation of canonical miR-3144-3p expression after ADAR1 editing (Fig. 4a). Since it edits the miR-3144-3p seed region, ADAR1 induces the re-expression of the target gene regulated by canonical miR-3144-3p. To test this hypothesis, we identified targets of canonical miR-3144-3p using the target prediction program TargetScan (http://www.targetscan.org/). Through this integrated analysis strategy based on Mfuzz and gene expression pattern analyses, we identified MSI2, STXBP4, and SUV39H as candidate target genes of canonical miR-3144-3p (Supplementary Fig. 5a, b; Supplementary Table 3). Among these candidates, only MSI2 expression was inhibited in both liver cancer cell lines (Supplementary Fig. 6a, b).

In other experiments, ADAR1 editing of canonical miR-3144-3p created a novel miRNA in which adenine at Position 3 of the seed region was changed to guanine (ED_miR-3144(3_A < G)). On the basis of the expression of the newly edited and generated ED_miR-3144(3_A < G) target gene was reduced as liver cancer progressed, five candidates, INMT, GHR, GLYAT, SLC38A4, and HMGCS2, were identified through target prediction and expression pattern analyses (Supplementary Fig. 5c, d; Supplementary Table 3). When MIHA and SNU-449 cells were transfected with a ED_miR-3144 (3_A < G) mimic or pcDNA3.1_ADAR1-p110_wild in the presence of primary mir-3144, only the expression of the SLC38A4 candidate target gene was inhibited in both liver cancer cell lines (Supplementary Fig. 6c, d). Therefore, we predicted that MSI2, a Musashi RNA-binding protein, as a target of canonical miR-3144-3p and SLC38A4 (solute carrier family 38 member 4) is a target for edited ED_miR-3144(3_A < G) and performed additional functional studies with these protein in liver cancer.

First, the regulation of canonical miR-3144-3p on MSI2 in liver cancer was confirmed. Ectopic expression of a miR-3144-3p mimic suppressed MSI2, whereas cotransfection of an antisense miR-3144-3p (AS-miR-3144-3p) mimic rescued this effect in liver cancer cells (Fig. 4b, upper). Note that an ED_miR-3144(3_A < G) mimic did not suppress MSI2 in either western blot or luciferase assays, implying the specific targeting of MSI2 by canonical miR-3144-3p (Fig. 4b, middle and lower). In addition, ectopic expression of primary mir-3144-3p or ADAR1 knockdown significantly suppressed MSI2 in both western blot and luciferase assays in the same cells (Fig. 4c). Notably, MSI2 suppression by ectopic expression of primary mir-3144 was rescued by pcDNA3.1_ADAR1-p110_wild, an active ADAR1, whereas the mutant form of ADAR1 had no effects on miR-3144 expression in the same cells (Fig. 4d).

Recently, MSI2 was reported to be a cancer driver gene and postulated to be a possible effector in the development of cancer^18^. We assessed the differential expression of effector genes in three large cohorts of liver cancer patients with data deposited in the Catholic_mLIHC, TCGA_LIHC, and ICGC_LIRI datasets (Supplementary Table 4). The analyses indicated that *HMGA2*, *MKI67*, *HOXA9*, and *MET* were significantly overexpressed in the abovementioned cohorts. Through a qRT-PCR assay of liver cancer cells of these four genes, only the expression of *MET* was found to be regulated by MSI2 (Supplementary Fig. 7a, b). We then used Western blot analyses to confirm that when MSI2 was selectively suppressed, MET was also suppressed, and conversely, when MSI2 was overexpressed, the expression of MET was also increased in liver cancer cells (Fig. 4e). Finally, MET abundance was found to be significantly increased as a result of knocking down overexpressed MSI2 in liver cancer cells (Fig. 4f). These results indicated that aberrant expression of ADAR1 in liver cancer induced excessive editing of canonical miR-3144-3p to induce MSI2-dependent MET signaling in liver cancer.

### miR-3144-3P editing induces MSI2 and concomitantly suppresses SLC38A4 expression in liver cancer

**Fig. 5:**
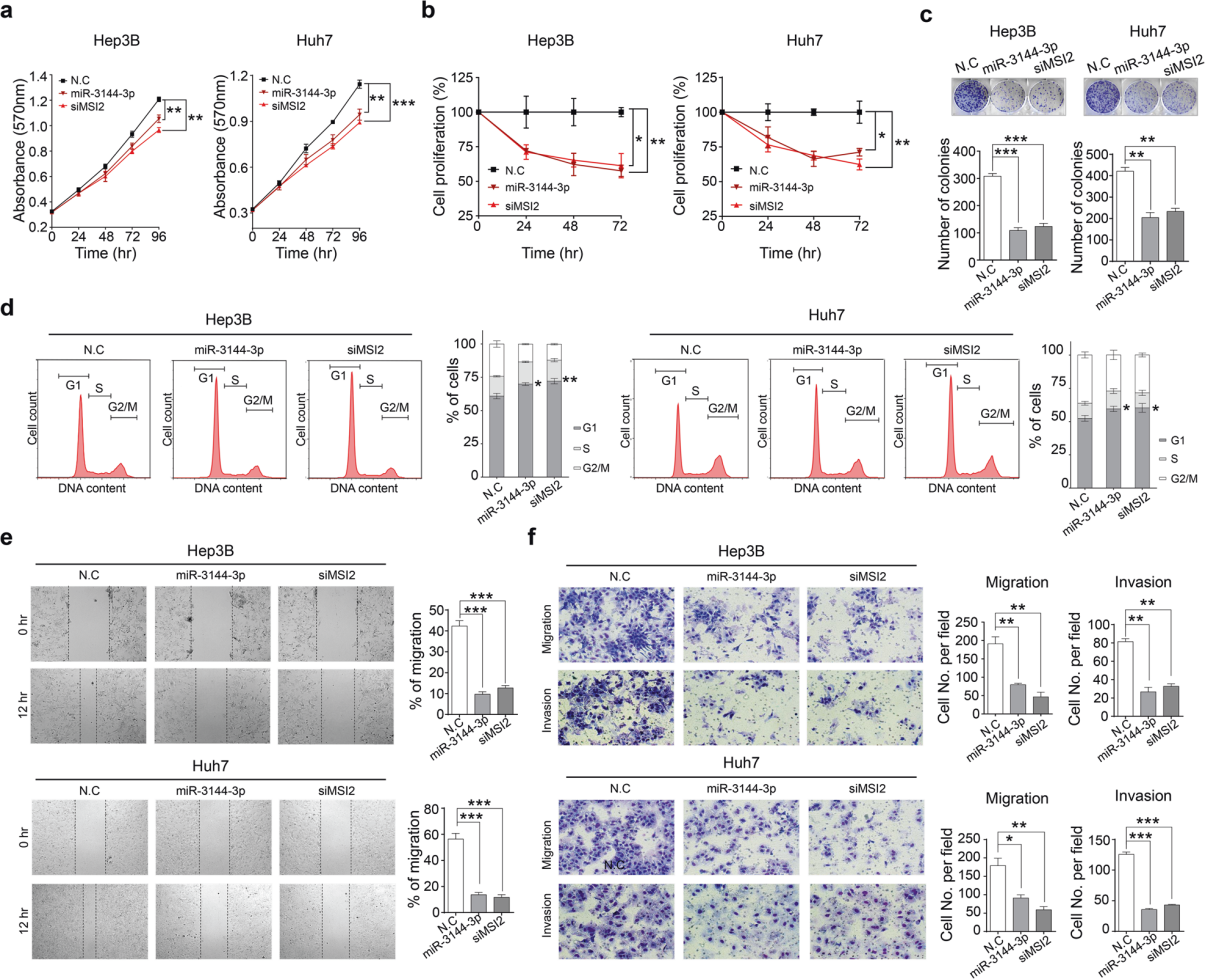
ADAR1-dependent canonical miR-3144-3p editing contributes to the tumorigenic potential of liver cancer. To assess the antitumorigenic effect of miR-3144-3p and MSI2 in liver cancer cells, MTT (**a**), BrdU (**b**), and clonogenic assays (**c**) were performed with miR-3144-3p mimic- or MSI2-siRNA (siMSI2)-treated cells. **d** The DNA content of PI-stained cells was analyzed by flow cytometry. The stained cell number to total cell number ratios are presented in the bar graph. **e** Representative cell image (left) and percentage (right) of migrated cells measured by scratch wound-healing assay. **f** Transwell migration and invasion assays. All data are shown as the mean ± SEM; *P < 0.05, **P < 0.01, ***P < 0.001 by unpaired Student’s *t*-test.

Because we found that excessive canonical miR-3144-3p editing by ADAR1 contributed to the malignant behavior of liver cancer cells, in vitro hepatocyte tumorigenesis experiments were performed to elucidate the tumor suppressive role played by canonical miR-3144-3p in liver cancer. The ectopic expression of a canonical miR-3144-3p mimic significantly repressed both the tumor growth and proliferation of liver cancer cells, and MSI2 knockdown exerted similar effects on the same cells (Fig. 5a–c). Both the canonical expression of miR-3144-3p and MSI2 knockdown were associated with G1/S phase arrest in the liver cancer cells, as determined via flow cytometry analyses of PI-stained liver cancer cells (Fig. 5d). In addition, the canonical expression of miR-3144-3p and MSI2 knockdown significantly suppressed not only wound-healing efficacy but also the migratory and invasive potential of the liver cancer cells (Fig. 5e, f).

**Fig. 6:**
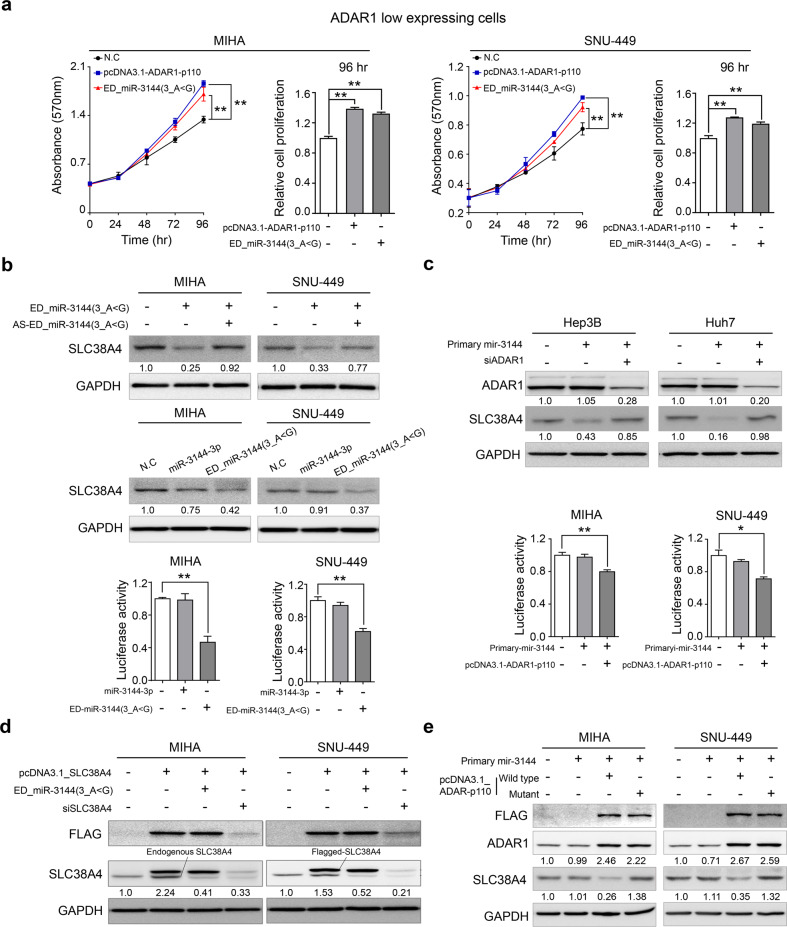
ED_miR-3144(3_A < G) functions as an onco-miR in liver cancer. **a** Cell growth was measured by MTT assay after transfection. **b** Cells were transfected with the ED_miR-3144(3_A < G) mimic or cotransfected with the mimic and antisense ED_miR-3144(3_A < G) (AS-ED_miR-3144(3_A < G)) (upper). miR-3144-3p or the ED_miR-3144(3_A < G) mimic was ectopically transfected into cells (middle). Western blot (middle) and luciferase reporter assays (lower). **c** Western blotting was performed after transfection of primary mir-3144 or cotransfection of miR-3144 with siADAR1 into Hep3B and Huh7 cells (upper). A luciferase reporter assay was performed with MIHA and SNU-449 cells (lower). **d** Western blot analysis showing the direct regulatory effect of ED_miR-3144(3_A < G) on SLC38A4 expression. **e** Cells were cotransfected with wild-type SLC38A4 or a pcDNA3.1_SLC38A4 mutant with primary mir-3144. All data are shown as the mean ± SEM; *P < 0.05, **P < 0.01 by unpaired Student’s *t*-test.

The edited product of canonical miR-3144-3p, ED_miR-3144(3_A < G), is a novel and nonannotated miRNA. Therefore, it was important to elucidate the functional roles played by ED_miR-3144(3_A < G) in liver cancer. Our analyses indicated that ED_miR-3144(3_A < G) was produced during liver cancerogenesis, and we speculated that this mutant miRNA suppresses liver cancer growth because it inhibits the translation of the mRNA of a specific gene. To test this hypothesis, we measured cell growth and differentiation rates after active ADAR1 (pcDNA3.1_ADAR1-p110) or ED_miR-3144(3_A < G) mimic was expressed in liver cancer cells with initial low ADAR1 expression. The ectopic expression of either ADAR1 or the ED_miR-3144(3_A < G) mimic significantly augmented the growth and proliferation rates of the liver cells (Fig. 6a). Next, we aimed to confirm our finding that the potential target of ED_miR-3144(3_A < G) is SLC38A4 in liver cancer cells. The ectopic expression of the ED_miR-3144(3_A < G) mimic suppressed SLC38A4 protein expression, whereas an antisense ED_miR-3144(3_A < G) mimic rescued SLC38A4 expression in liver cancer cells (Fig. 6b, upper). Notably, a canonical miR-3144-3p mimic did not affect SLC38A4 protein expression (Fig. 6b, middle), and similar results were obtained with a luciferase assay performed with the same cells (Fig. 6b lower). Moreover, a luciferase assay revealed the binding affinity of ED_miR-3144(3_A < G) for the SLC38A4 3’-UTR. In addition, the ectopic expression of a primary mir-3144 mimic suppressed SLC38A4 protein expression, whereas cotransfection of ADAR1 siRNA with the mimic rescued SLC38A4 expression in liver cancer cells (Fig. 6c). After transfection of a plasmid expressing SLC38A4 without the 3’-UTR and then transfection with the ED_miR-3144(3_A < G) mimic, we found that only endogenous SLC38A4 was selectively suppressed, whereas SLC38A4 siRNA transfection inhibited endogenous and ectopic SLC38A4, implying selective regulation of SLC38A4 mediated by ED_miR-3144(3_A < G) in liver cancer cells (Fig. 6d). These results were confirmed by a finding showing that SLC38A4 was inhibited when primary mir-3144 was cotransfected with pcDNA3.1-ADAR1-p110, whereas the pcDNA3.1-ADAR1-p110 mutant did not exert this inhibitory effect (Fig. 6e). These results showed that ADAR1 editing of canonical miR-3144-3p caused off-target effects on MSI2 expression, and in contrast, the newly created ED_miR-3144(3_A < G) mutant inhibited the expression of a different target, namely, SLC38A4, contributing to liver cancer. Notably, we observed a significant positive correlation between *MSI2* expression and *ADAR1* expression but a negative correlation between *SLC38A4* expression reported in publicly available (TCGA_LIHC, ICGC_LIRI, and GSE77314) and our multistage liver cancer (Catholic_mLIHC; GSE114564) datasets (Supplementary Fig. 8).

### In vivo functional validation of the effects on ADAR1, MSI2, and SLC38A4 in liver cancer

**Fig. 7:**
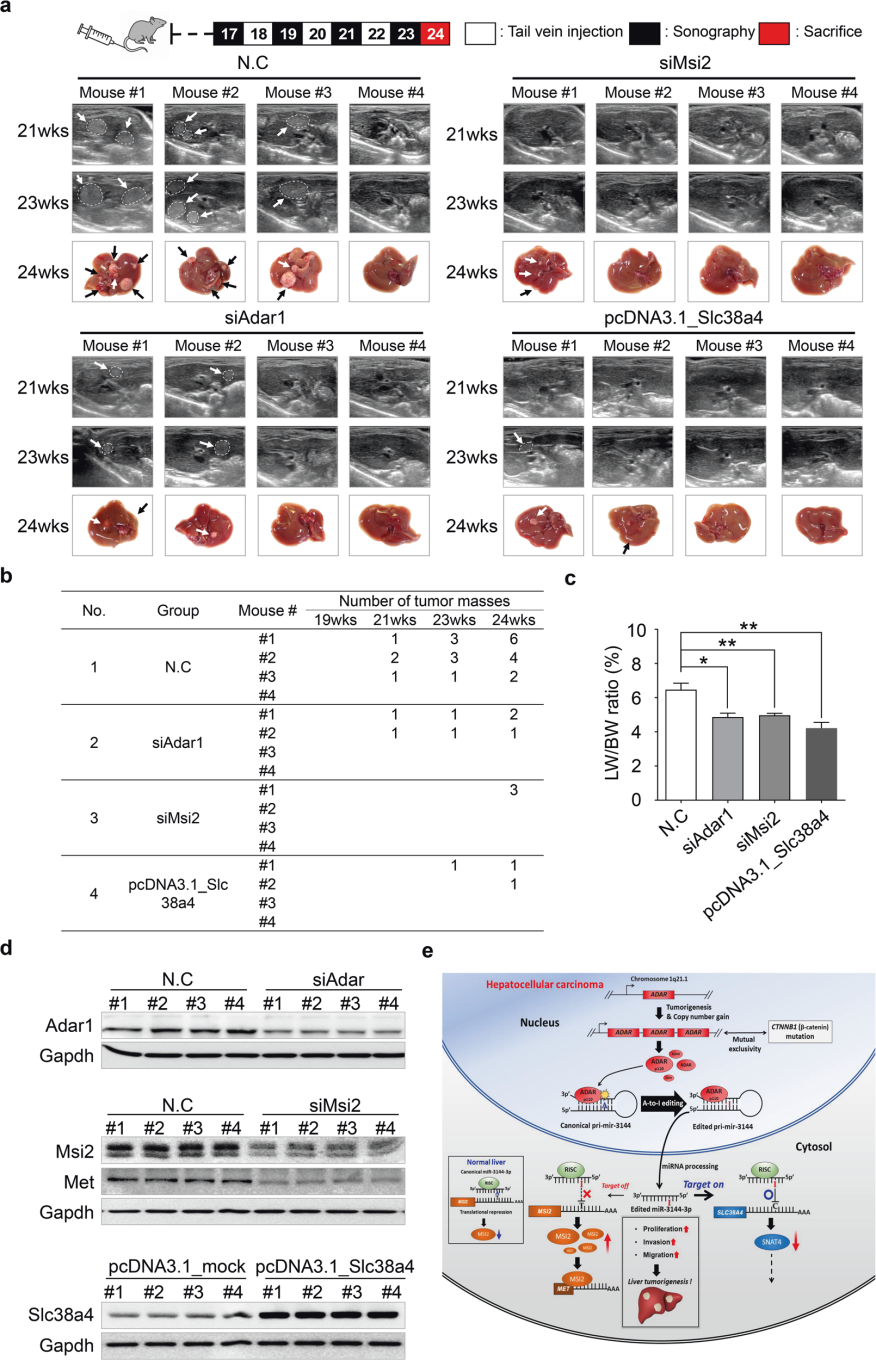
In vivo validation of ADAR1, MSI2, and SLC38A4 in mice. **a** Timeline of in the in vivo transfection of siAdar1, siMsi2, and pcDNA3.1_Slc38a4 in an H-*ras*-transgenic mouse model (upper). Representative ultrasonography images of the mouse liver cancer models at 21 and 23 weeks of age. Liver images taken at 24 weeks of age (lower). **b** The number of tumor masses in each mouse at the indicated weeks of age are listed in the table. **c** Bar chart showing the liver weight (LW) and body weight (BW) ratio (%) in each group. **d** Western blot analysis showing Adar1, Msi2, Met, and Slc38a4 expression in the H-*ras*-transgenic mice. Gapdh was used as the loading control. All data are shown as the mean ± SEM; *P < 0.05, **P < 0.01 by unpaired Student’s *t*-test. **e** Schematic showing the miR-3144-3p on target and off-target mechanisms induced by A-to-I RNA editing of upregulated ADAR1-p110 during liver cancer development.

Next, to demonstrate whether individual modulation of ADAR1, MSI2, and SLC38A4 affects liver tumorigenesis in vivo, we prepared H-*ras-*transgenic mice that spontaneously develop liver cancer beginning at ~14 weeks of age^13^. To investigate the cancer-preventive effect induced by targeting Adar1 and Msi2 or by re-expressing Slc38a4 in vivo, mice were administered Invivofectamine (siAdar1 and siMsi2) and Turbofect (pcDNA3.1_Slc38a4), both of which specifically target liver cells, via intravenous injections once per week beginning at 14 weeks of age (Fig. 7a, upper). Liver tumor masses were detectable at 21 weeks of age in the negative control (N.C.) group, with 3 of 4 mice developing large and multiple tumor masses; in contrast, whereas relatively fewer and smaller tumor masses were found in the siAdar1, siMsi2, and pcDNA3.1_Slc38a4 groups compared to those in the control group (Fig. 7a, lower). In addition, the incidence of mouse liver tumors in the negative control group was much higher than that in the siAdar1, siMsi2, or pcDNA3.1_Slc38a4 groups (Fig. 7b). Total liver weight changes were consistent with the marked inhibitory effect realized by targeting both Adar1 and Msi2 or by re-expressing Slc38a4, as indicated by the in vivo tumor load (Fig. 7c). Western blot analyses confirmed the modulation of Adar1, Msi2, and Slc38a4 expression in the noncancerous liver tissues surrounding tumor masses. Additionally, Met expression was repressed in the livers of siMsi2-treated mice, showing a downstream effect of Msi2 on Met expression in liver cancer in vivo (Fig. 7d).

## Discussion

Over the past few years, the effects of novel post-transcriptional regulatory mechanisms of action, including miRNA editing and chemical modifications, on miRNAs have been characterized, with important roles in cancer identified^19^. In particular, A-to-I editing of RNA, a widespread post-transcriptional process that increases various types of proteins and diverse functions from a limited set of genes, has recently emerged as an important mechanism in cancer biology. In addition to A-to-I editing of messenger RNA, some miRNA precursors undergo A-to-I editing, influencing the expression and/or function of mature miRNAs. For example, A-to-I editing in a recognition site of microprocessors such as DROSHA, DGCR8, and DICER may interfere with the biogenesis of mature miRNA^20^ or alter the recognition of the target mRNAs, particularly when a seed sequence in the miRNA is mutated by editing. Thus, editing may transform certain tumor suppressor miRNAs into oncogenic miRNAs^7^. In this study, including frequency and hotspot analyses using multistage liver cancer RNA genome data, miR-3144-3p was found to be closely associated with liver cancer. Through functional analyses, excessive editing of canonical miR-3144-3p was identified as a factor in inducing MSI2 expression, and at the same time, ED_miR-3144(3_A < G) generated by editing canonical miR-3144-3p inhibited SLC38A4, revealing correlations between miR-3144-3p editing with molecular drivers and signaling pathways in liver cancer.

A previous study into RNA-edited hotspots in miRNAs across cancer types revealed 19 ADAR-dependent A-to-I RNA-editing hotspots in the mature sequence of miRNAs, including miR-3144-3p^7^. These researchers subsequently focused on miR-200b and showed that edited miR-200b promoted cell invasion and migration because its ability to inhibit *ZEB1/ZEB2* was impaired and because it acquired the ability to repress a new target, *LIFR*, a metastatic suppressor. In our study, we identified miR-3144-3p as liver-cancer-specific ADAR1 expression that depends on an edited miRNA, specifically revealing that the adenine at Position 3 in miR-3144-3p is an editing hotspot (Supplementary Fig. 3). In addition, we found a frequent miR-200b editing event at Position 5 of mature miRNA, as reported in a previous study of liver cancer. However, no significant change in the expression of miR-200b was found in liver cancer tissues compared with that in normal tissues, whereas the expression of miR-3144-3p was increased ~20-fold or more in the liver cancer tissues (data not shown). In addition, our results showed that the frequency of miR-3144-3p editing increased liver cancer progression, which was accompanied by an increase in the gene copy number of corresponding loci, implying that miRNA editing contributes to liver cancer pathology.

Dysregulation of miR-3144-3p was first reported in human liver cancer, but few functional studies have been completed to date^21^. The original function attributed to Musashi (MSI) RNA-binding proteins was found to be regulation of asymmetric cell division during embryonic development, and many studies have reported that MSI2 is closely associated with advanced clinical stages of several cancers, including liver cancer, but the signaling pathways that regulate MSI2 expression are currently unknown^18^. Additionally, even though MSI2 target genes, such as MYC, LIN28A, and MET, have been extensively studied, the upstream mechanism driving MSI2 overexpression in liver cancer development remains unclear. Our data demonstrated that canonical miR-3144-3p plays a role in regulating the translation of *MSI2* mRNA in normal hepatocytes, although excessive editing of canonical miR-3144-3p is induced by the aberrant regulation and activity of ADAR1. We also found that the loss of miR-3144-3p induces MSI2 overexpression and contributes to liver cancer.

The solute carrier protein (SLC) superfamily member SLC38A4 is a system A amino acid transporter. System A is a ubiquitous Na^+^-dependent transporter that converts zwitterionic amino acids into N-methylated amino acids, such as alanine, serine, and glutamine^22^. Amino acids are required for the survival and growth of highly proliferative cells such as embryonic cells and cancer cells. However, SLC38A4 has been reported to function as a tumor suppressor in liver cancer through its modulation of Wnt/β-catenin/MYC/HMGCS2 axis acativation^23^. Our analyses showed the downregulation of SLC38A4 in large cohorts of liver cancer patients (Supplementary Table 3). In addition, Kaplan‒Meier survival analyses with a large cohort of liver cancer patients (the TCGA_LIHC dataset) showed that the 5-year overall survival rate of liver cancer patients with low SLC38A4 expression was significantly lower than that of patients with high SLC38A4 expression (data not shown). Transfection with a Slc38a4 expression plasmid significantly suppressed the tumorigenicity of an H-*ras* transgenic mouse liver cancer model. (Fig. 7a, b). Together, these findings indicated that the SLC38A4 tumor suppressor was inhibited by the ED_miR-3144(3_A < G) mutant generated by excessive ADAR1-dependent miR-3144-3p editing and that these effects contribute to the malignant transformation and growth of liver cancer cells.

Our results demonstrate that excessive ADAR1-dependent editing of canonical miR-3144-3p plays a pivotal role in the development and progression of liver cancer. Maintaining the normal activity and expression of ADAR1 appears to be important in maintaining the balance of canonical miRNAs that function in mitogenic signaling in hepatocytes. Aberrant overexpression of ADAR1 induces editing of canonical miR-3144-3p to induce translation of the *MSI2* oncogene, thereby augmenting the growth, proliferation, motility, and invasive potential of hepatocytes. In addition, ADAR1-dependent excessive editing of canonical miR-3144-3p leads to the generation of a novel ED_miR-3144(3_A < G) mutant that specifically suppresses *SLC38A4* mRNA translation to inactivate the tumor suppressor function of SLC38A4 during liver cancer progression (Fig. 7e). Together, these findings identify a central role for ADAR1-dependent miR-3144-3p editing in liver cancer and suggest its potential therapeutic value for the treatment of liver cancer.

## Supplementary information

Supplementary Material
