# Supplementary material for: ADAR1-dependent miR-3144-3p editing simultaneously induces MSI2 expression and suppresses SLC38A4 expression in liver cancer
Source: Exp Mol Med. 2023 Jan 4;55(1):95–107. doi: 10.1038/s12276-022-00916-8 (PMC9898302; doi:10.1038/s12276-022-00916-8)
Supplement: Supplementary file 1 — Supplementary Material [file 12276_2022_916_MOESM1_ESM.pdf]

ADAR1-dependent miR-3144-3p editing simultaneously induces MSI2 expression and suppresses SLC38A4 expression in liver cancer

Hyung Seok Kim,[1,6] Min Jeong Na,[1,2,3,6] Keun Hong Son,[4] Hee Doo Yang,[2,5] Sang Yean Kim,[2,5] Eunbi Shin,[1,2,3] Jin Woong Ha,[1,2,3] Soyoung Jeon,[1,2,3] Keunsoo Kang,[4] Kiho Moon,[5] Won Sang Park[1,2] and Suk Woo Nam.[1,2,3,5]

[1]Department of Pathology, College of Medicine, The Catholic University of Korea, 222 Banpo-daero, Seocho-gu, Seoul 06591, Republic of Korea.

[2]Functional RNomics Research Center, The Catholic University of Korea, 222 Banpo-daero, Seocho-gu, Seoul 06591, Republic of Korea.

[3]Department of Biomedicine & Health Sciences, Graduate School, The Catholic University of Korea, Seoul, 06591, Korea.

[4]Department of Microbiology, Dankook University, Cheonan 31116, Republic of Korea.

[5]NEORNAT Inc., Rm. #5104 Bldg. A, Omnibus Park, 222 Banpo-daero, Seocho-gu, Seoul 06591, Republic of Korea.

[6]These authors contributed equally: Hyung Seok Kim and Min Jeong Na.

Correspondence

Suk Woo Nam, Ph.D., Department of Pathology, College of Medicine, The Catholic University of Korea, 222 Banpo-daero, Seocho-gu, Seoul 0659, Republic of Korea. email: swnam@catholic.ac.kr

Supplementary tables

Supplementary Table 1. Differential expression of RNA editing gene families in liver cancer.

Family	No.	Gene	TCGA_LIHC*			Catholic_mLIHC**			ICGC_LIRI***			GSE77314		
			Non-tumor (n=50)	Tumor (n=371)	Fold change	Normal (n=15)	Tumor (n=56)	Fold change	Non-tumor (n=202)	Tumor (n=238)	Fold change	Non-tumor (n=50)	Tumor (n=50)	Fold change
ADAR	1	**ADAR1**	**16.43**	**33.55**	**2.04**	**46.65**	**73.67**	**1.58**	**21.24**	**41.38**	**1.95**	**25.44**	**41.12**	**1.62**
	2	ADARB1	0.67	1.02	1.53	4.93	3.76	-1.31	2.40	2.71	1.13	3.62	2.83	-1.28
	3	ADARB2	0.04	0.06	1.35	0.23	0.20	-1.14	N.E	0.12	-	0.27	0.14	-1.96
APOBEC	1	AICDA	N.E	N.E	-	N.E	N.E	-	N.E	N.E	-	N.E	N.E	-
	2	APOBEC1	N.E	N.E	-	N.E	N.E	-	N.E	0.46	-	N.E	N.E	-
	3	APOBEC2	N.E	0.15	-	N.E	N.E	-	0.17	0.22	1.32	0.10	0.16	1.70
	4	APOBEC3A	0.25	0.11	-2.20	1.68	0.51	-3.29	0.73	1.39	1.91	0.50	0.23	-2.16
	5	APOBEC3B	0.41	2.27	5.52	0.23	1.27	5.43	0.49	2.14	4.39	0.30	2.48	8.26
	6	APOBEC3C	2.20	3.54	1.20	1.97	2.07	1.05	3.17	3.02	-1.05	3.07	2.19	-1.40
	7	APOBEC3D	0.47	0.86	1.84	0.63	0.81	1.30	0.92	1.02	1.11	1.05	0.88	-1.19
	8	APOBEC3F	0.96	1.36	1.43	1.27	1.40	1.10	0.84	1.06	1.26	1.36	1.55	1.14
	9	APOBEC3G	0.71	1.07	1.52	2.88	2.85	-1.01	3.41	3.67	1.08	4.43	3.51	-1.26
	10	APOBEC3H	0.19	0.43	2.27	0.45	0.44	-1.02	0.91	1.13	1.24	1.06	0.86	-1.23
	11	APOBEC4	N.E	N.E	-	N.E	N.E	-	N.E	N.E	-	N.E	N.E	-

*TCGA_LIHC: The Cancer Genome Atlas_Liver Hepatocellular Carcinoma

**Catholic_mLIHC: Catholic university_multistage Liver Hepatocellular Carcinoma (GSE114564)

***ICGC_LIRI: International Cancer Genome Consortium_Liver Cancer-RIKEN.JP

NE: Non-expression

Supplementary Table 2. Systematic analysis of mutual exclusivity between *ADAR1* amplification and *CTNNB1* mutation in various cancers.

No.	Abbreviation	Study	Samples	CTNNB1	ADAR1	Both	Neither	p-value	Tendency
**1**	**LIHC**	**Liver hepatocellular carcinoma**	**366**	**94**	**41**	**5**	**226**	**0.004****	**Mutual exclusivity**
2	STAD	Stomach adenocarcinoma	393	27	18	6	342	0.01**	Co-occurrence
3	LUAD	Lung adenocarcinoma	230	5	33	4	188	0.039*	Co-occurrence
4	PRAD	Prostate adenocarcinoma	492	13	11	2	466	0.056	None
5	SKCM	Skin Cutaneous Melanoma	287	16	12	3	256	0.067	None
6	UCS	Uterine Carcinosarcoma	56	0	4	1	51	0.089	None
7	UCEC	Uterine Corpus Endometrial Carcinoma	242	70	17	3	152	0.094	None
8	OV	Ovarian serous cystadenocarcinoma	311	5	26	2	278	0.124	None
9	PAAD	Pancreatic adenocarcinoma	149	4	8	1	136	0.271	None
10	CESC	Cervical squamous cell carcinoma	191	7	10	1	173	0.384	None
11	ACC	Adrenocortical carcinoma	88	14	2	1	71	0.433	None
12	ESCA	Esophageal carcinoma	184	10	9	1	164	0.469	None
13	BRCA	Breast invasive carcinoma	963	5	112	1	845	0.528	None
14	BLCA	Bladder Urothelial Carcinoma	127	6	13	1	107	0.568	None
15	COADREAD	Coloreactal adenocarcinoma	220	12	7	0	201	0.671	None
16	KIRC	Kidney renal clear cell carcinoma	448	51	9	1	387	0.673	None
17	LUSC	Lung squamous cell carcinoma	178	5	11	0	162	0.724	None
18	HNSC	Head and Neck squamous cell carcinoma	504	10	14	0	480	0.753	None
19	SARC	Sarcoma	243	5	12	0	226	0.775	None
20	PCPG	Pheochromocytoma and Paraganglioma	162	1	6	0	155	0.963	None
21	LGG	Brain Lower Grade Glioma	283	2	4	0	277	0.972	None
22	THYM	Thymoma	123	3	1	0	119	0.976	None
23	KIRP	Kidney renal papillary cell carcinoma	280	2	3	0	275	0.979	None
24	THCA	Thyroid carcinoma	399	2	1	0	396	0.995	None
25	LAML	Acute Myeloid Leukemia	188	0	0	0	188	1	None
26	CHOL	Cholangiocarcinoma	35	0	5	0	30	1	None
27	GBM	Glioblastoma multiforme	273	0	1	0	272	1	None
28	KICH	Kidney Chromophobe	66	2	0	0	64	1	None
29	DLBC	Lymphoid neoplasm	48	0	3	0	45	1	None
30	MESO	Mesothelioma	83	0	4	0	79	1	None
31	TGCT	Testicular Germ Cell Tumors	149	0	1	0	148	1	None
32	UVM	Uveal Melanoma	80	1	0	0	79	1	None

*The genomic alteration and mutation data are acquired from cBioPortal (www.cbioportal.org)

Supplementary Table 3. Differential expression and fold change of miR-3144-3p and ED_miR-3144(3_A<G) target candidates in liver cancer RNA-seq datasets.

			TCGA_LIHC			Catholic_mLIHC			ICGC_LIRI		
	No.	Genes	Non-tumor (n=50)	Tumor (n=371)	Fold change	Normal (n=15)	Tumor (n=56)	Fold change	Non-tumor (n=202)	Tumor (n=238)	Fold change
Canonical *miR-3144-3p* targets	1	STXBP4	0.12	0.43	3.48	3.49	6.61	1.89	0.37	0.89	2.37
	2	SUV39H2	0.69	1.89	2.74	2.25	5.21	2.31	1.07	2.32	2.16
	3	MSI2	0.56	1.44	2.56	14.56	25.21	1.73	1.75	3.44	1.96
Edited_*miR-3144(3_A<G)* targets	1	INMT	9.65	2.53	-3.82	26.95	9.6	-2.79	11	3.5	-3.14
	2	GHR	27.20	7.56	-3.60	84.66	26.75	-3.17	33.28	10.33	-3.22
	3	GLYAT	58.82	21.38	-2.75	153.18	37.14	-4.12	100.66	44.71	-2.25
	4	SLC38A4	114.84	60.33	-1.90	332.43	134.61	-2.47	133.36	75.11	-1.78
	5	HMGCS2	664.92	436.49	-1.52	826.03	347.58	-2.38	652.14	350.01	-1.86

Supplementary Table 4. Differential expression of MSI2 targeting genes in liver cancer.

		Catholic_mLIHC			TCGA_LIHC			ICGC_LIRI		
No.	Genes	Normal (n=15)	Tumor (n=56)	Fold change	Non-tumor (n=50)	Tumor (n=371)	Fold change	Non-tumorl (n=202)	Tumor (n=238)	Fold change
**1**	**HMGA2**	**0.07**	**1.15**	**16.08**	**0.003**	**0.16**	**51.56**	**0.17**	**1.68**	**9.65**
**2**	**MKI67**	**0.70**	**6.69**	**9.53**	**0.20**	**1.57**	**7.81**	**0.39**	**3.29**	**8.36**
**3**	**HOXA9**	**0.03**	**0.13**	**3.68**	**0.008**	**0.07**	**8.24**	**0.05**	**0.19**	**3.50**
**4**	**MET**	**37.51**	**73.57**	**1.96**	**3.53**	**3.96**	**1.12**	**10.64**	**19.11**	**1.80**
5	SMAD3	8.45	11.33	1.34	1.83	2.55	1.39	7.70	12.01	1.56
6	BECN1	23.09	27.53	1.19	2.01	2.47	1.23	14.01	23.86	1.70
7	TGFBI	95.44	106.02	1.11	3.82	4.58	1.20	64.99	96.52	1.49
8	LFNG	1.34	1.33	-1.004	0.79	1.27	1.61	1.76	3.66	2.08
9	NOTCH1	1.79	1.77	-1.01	1.39	1.80	1.29	2.64	3.50	1.33
10	IKZF2	3.84	3.74	-1.03	0.22	0.23	1.08	0.65	0.57	-1.12
11	BMPR1A	6.60	6.42	-1.03	0.77	1.09	1.42	2.22	3.57	1.61
12	PTEN	67.91	64.41	-1.05	2.69	2.72	1.01	9.94	10.65	1.07
13	KRAS	10.93	10.00	-1.09	1.76	1.97	1.12	4.89	5.60	1.15
14	BRD4	11.79	10.10	-1.17	2.34	2.79	1.20	6.65	7.97	1.20
15	NUMB	33.57	27.61	-1.22	2.14	2.33	1.09	17.16	18.52	1.08
16	LRIG1	15.95	11.66	-1.37	2.48	2.60	1.05	7.90	8.78	1.11
17	AHR	59.56	42.20	-1.41	3.59	3.31	-1.09	13.55	15.95	1.18
18	FLT3	0.50	0.34	-1.45	0.21	0.15	-1.48	0.38	0.27	-1.39
19	CDKN1A	35.99	22.39	-1.61	5.33	5.18	-1.03	65.02	54.62	-1.19
20	MYC	28.82	15.19	-1.90	4.40	3.60	-1.22	24.54	23.86	-1.03
21	AR	29.92	13.50	-2.22	3.26	2.35	-1.39	5.16	3.58	-1.44
22	ESR1	63.15	14.62	-4.32	1.60	0.49	-3.27	4.03	1.28	-3.14
23	LIN28A	0	0.008	-	0.001	0.04	35.46	0.03	0.51	16.56

Supplementary Table 5. List of siRNA, miRNA mimics, and antisense miRNA sequences used in transfection.

siRNA/miRNA	Accession No.	Strand	Nucleotide sequence
Control siRNA		Sense	5'-CCUACGCCACCAAUUUCGU-3'
		Antisense	5'-ACGAAAUUGGUGGCGUAGG-3'
ADAR1 siRNA	NM_001025107	Sense	5'-CACCAAGGGAAGUUGACUA-3'
		Antisense	5'-UAGUCAACUUCCCUUGGUG-3'
MSI2 siRNA	NM_138962	Sense	5'-CAGCCGAAAGAAGUCAUGU-3'
		Antisense	5'-ACAUGACUUCUUUCGGCUG-3'
SLC38A4 siRNA	NM_018018	Sense	5'-CAUUCUUGCUCACUUCUAU-3'
		Antisense	5'-AUAGAAGUGAGCAAGAAUG-3'
Adar1 siRNA	NM_019655	Sense	5'-AGACAGUGGUCAACCAGAA-3'
		Antisense	5'-UUCUGGUUGACCACUGUCU-3'
Msi2 siRNA	NM_054043	Sense	5'-GUUAGAUUCCAAGACGAUU-3'
		Antisense	5'-AAUCGUCUUGGAAUCUAAC-3'
miR-3144-3p	NR_036098	Sense	5’-AUAUACCUGUUCGGUCUCUUUA-3'
AS-miR-3144-3p		Antisense	5’-UAAAGAGACCGAACAGGUAUAU-3'
ED_miR-3144(3_A<G)		Sense	5’-AUGUACCUGUUCGGUCUCUUUA-3'
AS-ED_miR3144(3_A<G)		Antisense	5’-UAAAGAGACCGAACAGGUACAU-3'

Supplementary Table 6. List of primer sequence used in PCR experiments.

Gene	Accession No.	Primer	Nucleotide sequence
ADAR1	NM_001025107	Forward	5'-TGGCAGCCTCCGGGTG-3'
		Reverse	5'-TGTCTGTGCTCATAGCCTTG-3'
gADAR1	NM_001025107	Forward	5'-CTTTCCGTCAAGATTTAAATT-3'
		Reverse	5'-GGGATGATTCTTCTGATTTTC-3'
ADAR1-mutant	NM_001025107	Forward	5'-GAGAAACTGTCAATGACTGCTAT GCAGCAATAATCTCCCGGAGAGGCTT-3'
		Reverse	5'-AAGCCTCTCCGGGAGATTATTGCTG CATAGCAGTCATTGACAGTTTCTC-3'
MSI2	NM_138962	Forward	5'-AAAGGAGCGCCAGGGTTAAA-3'
		Reverse	5'-GTCTGCGAACGTGACGAAAC-3'
STXBP4	NM_178509	Forward	5'-CCGGAGCCAAGTTGAGGTTA-3'
		Reverse	5'-GGAGTGGATGAGGTCTTTGGG-3'
SUV39H2	NM_001193424	Forward	5'-GAGGCGCGAGGAGCTTG-3'
		Reverse	5'-GCAGTAACGGGCACTTCAGA-3'
GHR	NM_000153	Forward	5'-AGCGCAGACGCGAACC\-3'
		Reverse	5'-AGGCTCCTTAGAAGAATTTGTCTTT-3'
GLYAT	NM_005834	Forward	5'-CTGGCTGCATCAGGGAGAAA-3'
		Reverse	5'-GTCATATCCTGCTCCTGAGGG-3'
SLC38A4	NM_018018	Forward	5'-TGCAGGAAACTGTGATTTGC-3'
		Reverse	5'-TTCGAGCCCACCAACTTAAT-3'
HMGCS2	NM_005518	Forward	5'-CCACCTGGTGACACAAACAG-3'
		Reverse	5'-TATGATTCACGGGGAGAAGC-3'
INMT	NM_006774	Forward	5'-GAAACAGCGGCCGATGGGA-3'
		Reverse	5'-AGGCATCAAGGCTACAGCAG-3'
HMGA2	NM_003483	Forward	5'-TGGGAGGAGCGAAATCTAA-3'
		Reverse	5'-GGTGAACTCAAGCCGAAG-3'
MET	NM_000245	Forward	5'-TGCAGCGCGTTGACTTATTCATGG-3'
		Reverse	5'-GAAACCACAACCTGCATGAAGCGA-3'
HOXA9	NM_152839	Forward	5'-CCCCATCGATCCCAATAACCC-3'
		Reverse	5'-GGTGAGGTTGAGCAGTCGAG-3'
MKI67	NM_002417	Forward	5'-TTGGTACTGGGGGAGGGAGA-3'
		Reverse	5'-TGGGAGGCGAAAAAGTAAAA-3'
mir-3144	NR_036098	Forward	5'-TCATGCAAATGGAAACCAAA-3'
		Reverse	5'-CGTTCATTGTTAAAGGTCACGA-3'
GAPDH	NM_002046	Forward	5'-ACCAGGTGGTCTCCTCTGAC-3'
		Reverse	5'-TGCTGTAGCCAAATTCGTTG-3'
gGAPDH	NM_002046	Forward	5'-ACCCAGAAGACTGTGGATGG-3'
		Reverse	5'-TTCTAGACGGCAGGTCAGGT-3'

gADAR1, primers for amplifying genomic *ADAR1*

ADAR1-mutant, primers for generation of deaminase inactivated pcDNA3.1_ADAR1-p110

mir-3144, primers for sanger sequencing of mir-3144

Supplementary figures

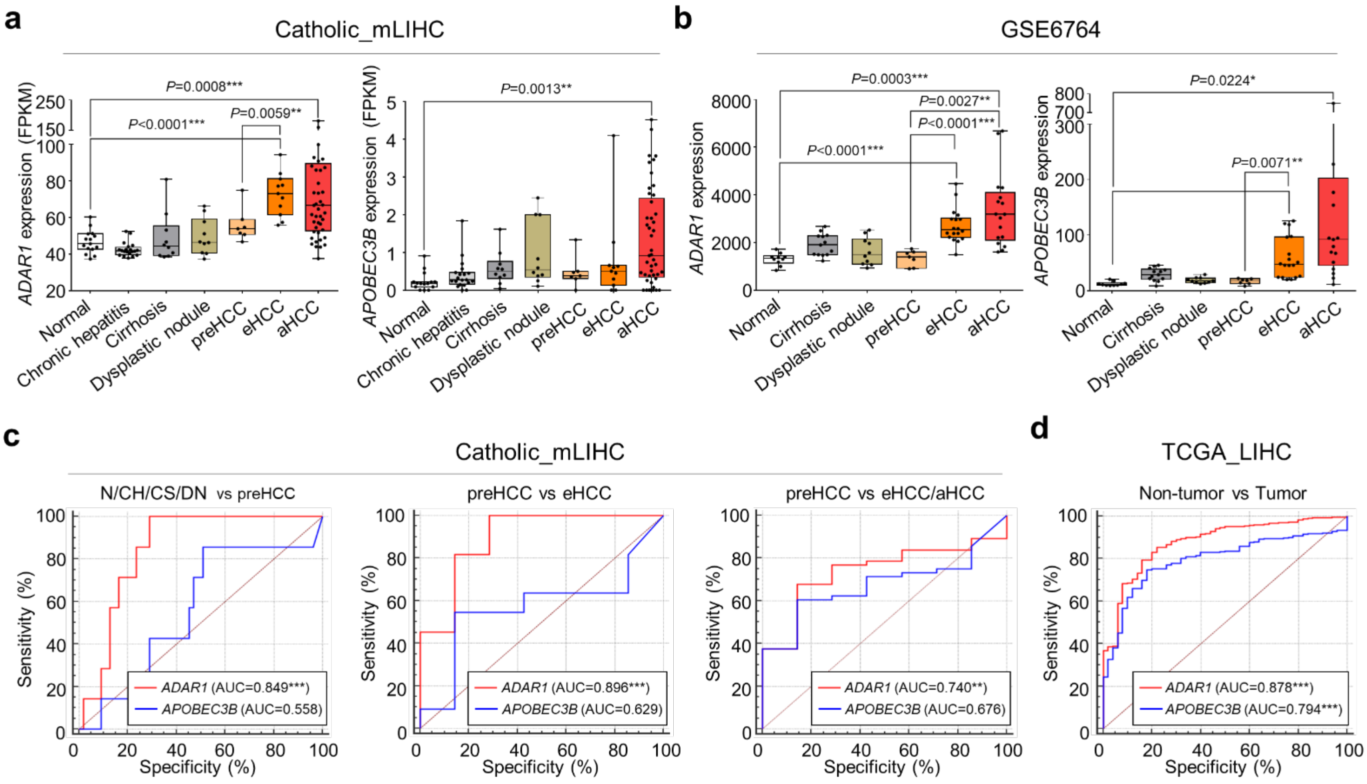

Supplementary Fig. 1. Comparison of ADAR1 and APOBEC3B expression in multi-stage liver cancer. ADAR1 and APOBEC3B expression in multi-stage liver cancer. Expression changes of ADAR1 and APOBEC3B in multi-stage liver disease patients of **a** Catholic_mLIHC (n=108) and **b** GSE6764 (n=17). Statistically significant differences were determined using Welch's *t* test, $P < 0.05$*, $P < 0.01$**, $P < 0.0001$***. The ROC curve analysis of the ADAR1 and APOBEC3B in liver cancer patients of **c** Catholic_mLIHC and **d** TCGA_LIHC. Statistically significant difference in AUC is compared with reference line (AUC=0.5). N, normal liver. CH, chronic hepatitis B virus infection. DN, dysplastic nodule.

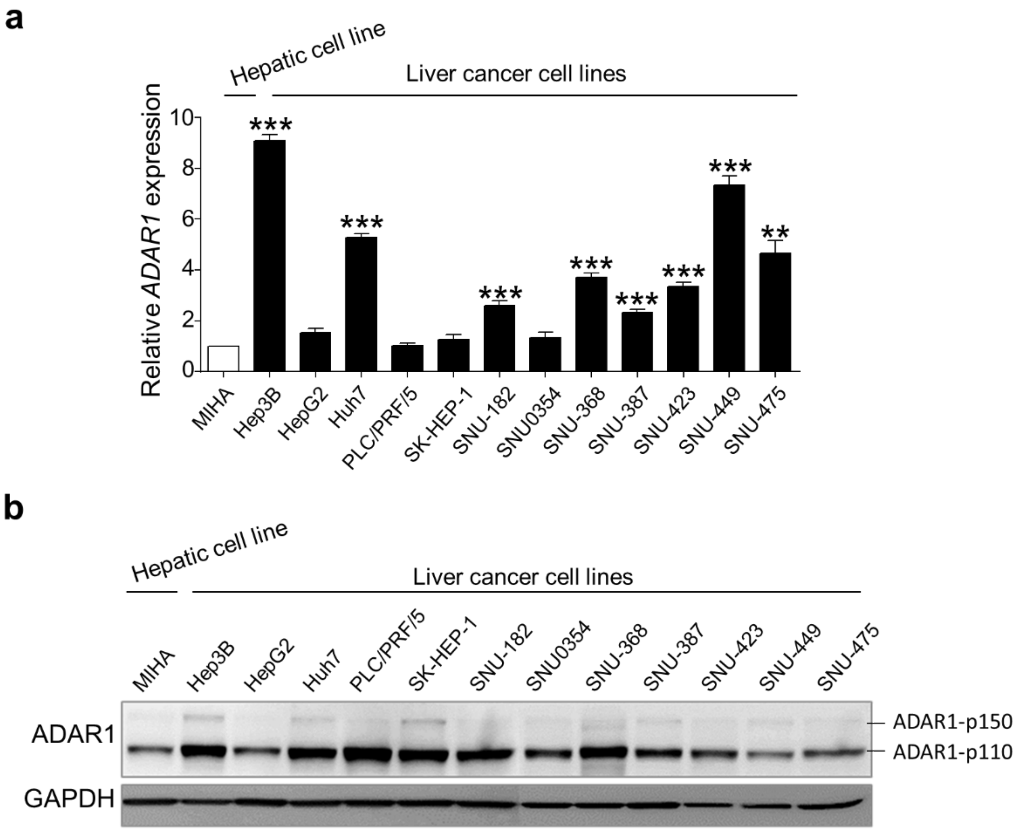

Supplementary Fig. 2. ADAR1 expression in liver cancer cell lines. ADAR1 is overexpressed in liver cancer cell lines. Expression of ADAR1 in an immortalized normal hepatic cell line and twelve liver cancer cell lines using qRT-PCR **a** and Western blot analysis **b**. **P* < 0.05, ***P* < 0.01, ****P* < 0.001; unpaired *t*-test.

a

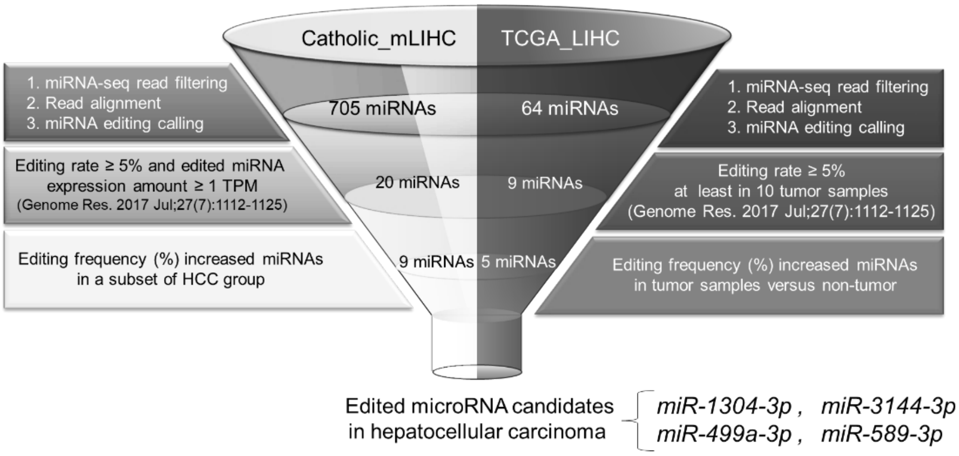

b

miRNAs	Edited position	Mature miRNA sequence
hsa-miR-1304-3p	5	ucuc<u>A</u>cuguagccucgaacccc
**hsa-miR-3144-3p**	**3**	**au<u>A</u>uaccuguucggucucuuua**
hsa-miR-499a-3p	4	aac<u>A</u>ucacagcaagucugugcu
hsa-miR-589-3p	6	ucaga<u>A</u>caaaugccgguucccaga

c

Edited miRNA candidates in HCC	Catholic_mLIHC Editing frequency (%) (Editing level >0%)				TCGA_LIHC Editing frequency (%) (Editing level ≥5%)	
	Normal (n=15)	preHCC (n=7)	eHCC (n=11)	aHCC (n=45)	Non-tumor (n=50)	Tumor (n=372)
hsa-miR-1304-3p	0.0	0.00	9.09	7.0	0.0	3.8
**hsa-miR-3144-3p**	**0.0**	**28.57**	**18.18**	**32.6**	**0.0**	**12.6**
hsa-miR-499a-3p	0.0	0.00	0.00	2.3	0.0	3.5
hsa-miR-589-3p	26.7	0.00	27.27	55.8	96.0	97.8

d e

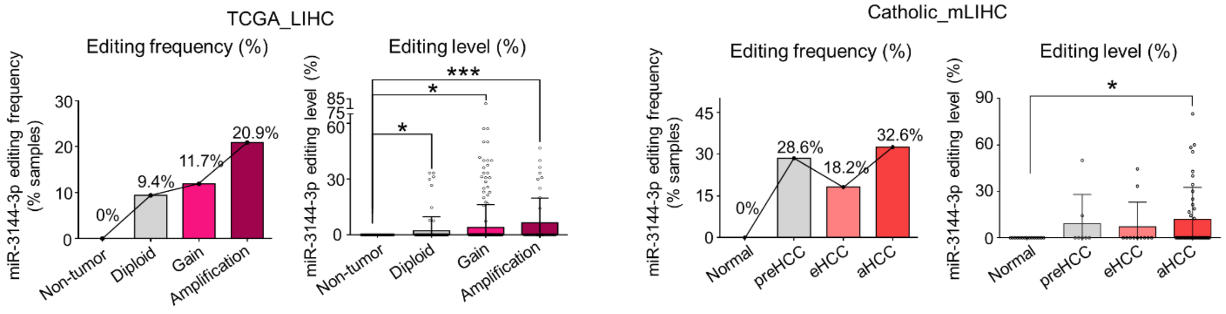

Supplementary Fig. 3. Identification of ADAR1 editing miRNAs in liver cancer. Identification of edited miRNA hotspots in liver cancer. **a** Flowchart for the bioinformatic pipeline for identification of edited miRNAs in liver cancer. **b** Editing position summary of the 4 edited miRNAs in liver cancer. **c** The A-to-I editing frequency of the 4 miRNAs was analyzed with the two liver cancer small-RNA sequencing datasets of Catholic_mLIHC and TCGA_LIHC. **d** Copy gain and amplification of *ADAR1* are associated with increased editing frequency (% samples) of miR-3144-3p compared to normal and diploid groups analyzed with liver cancer TCGA dataset (left panel). Genomic copy number gain (gain and amplification) is related to the increased editing level (%) of miR-3144-3p shown as a boxplot (right panel). **e** The editing frequency (% patient samples) of miR-3144-3p is increased in overt liver cancer (left panel). Differential editing level (%) of miR-3144-3p in multi-stage human liver cancer tissues analyzed with Catholic_mLIHC (right panel).

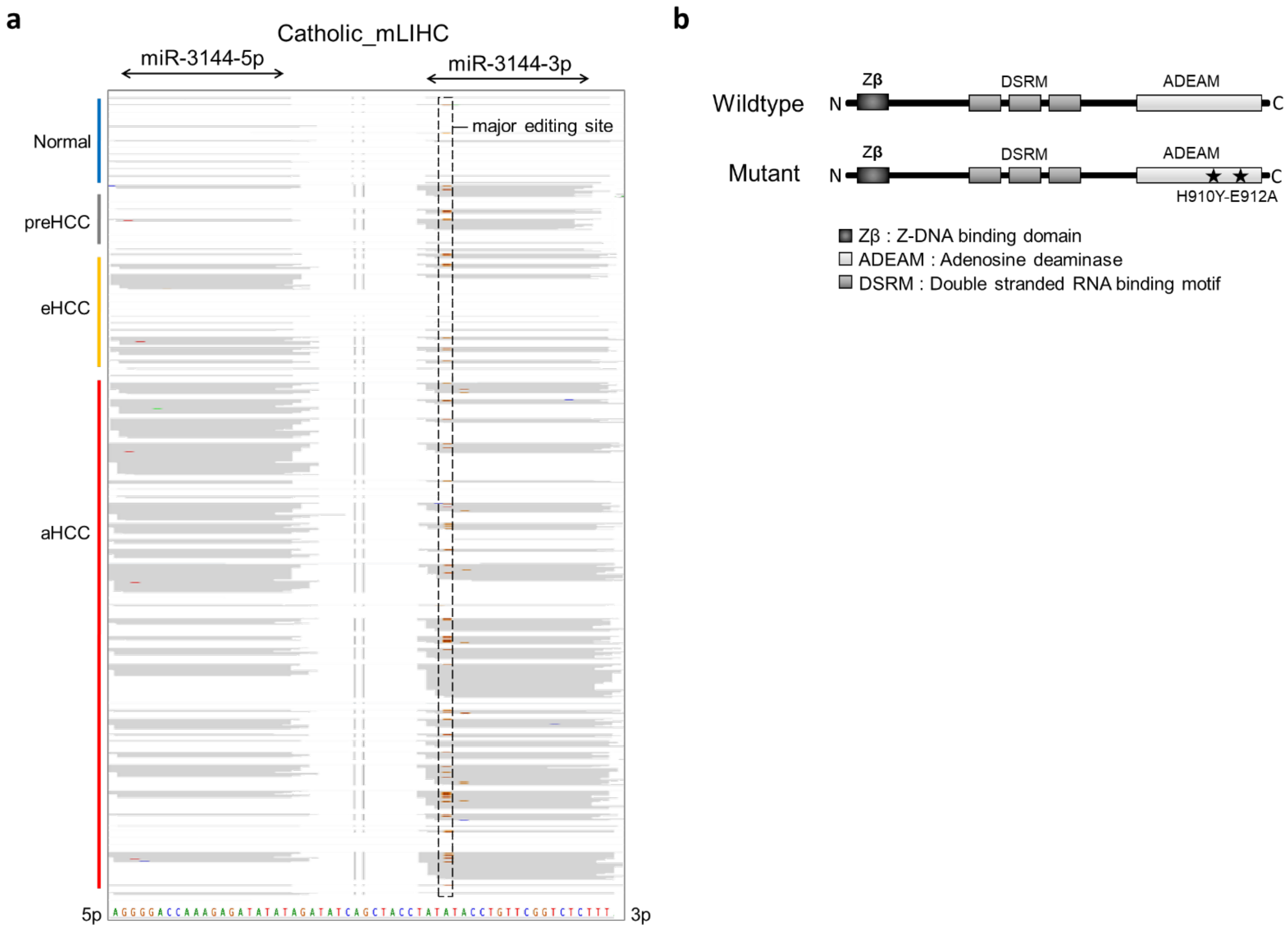

Supplementary Fig. 4. Visualization of miR-3144-3p editing *in silico*. Validation of miR-3144-3p editing *in silico* and *in vitro*. **a** The A-to-I editing sites of miR-3144-3p seed region are observed in overt liver cancer patient samples visualized by Integrative genomics viewer (IGV). **b** Schematic protein structure diagram of wildtype and adenosine deaminase mutant for ADAR1-p110.

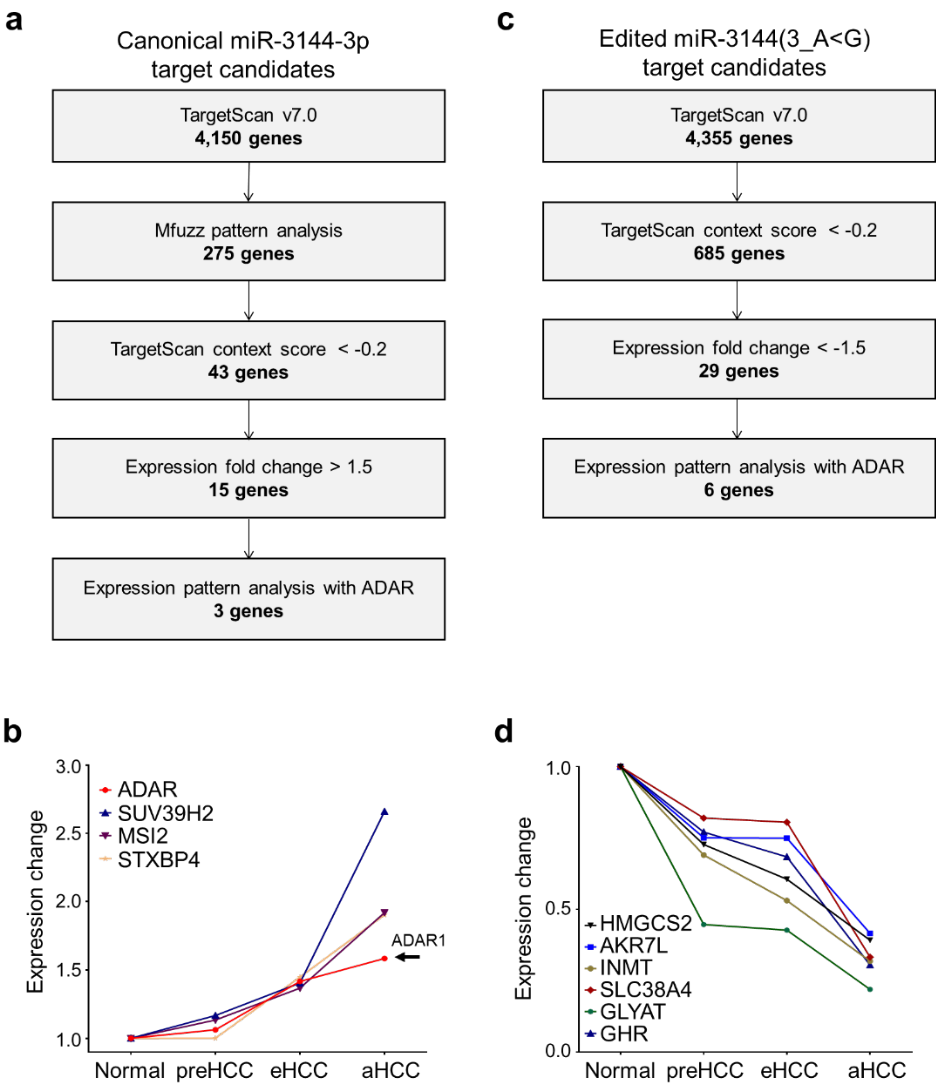

Supplementary Fig. 5. Strategies to identify targets of canonical miR-3144-3p and Edited miR-3144(3_A<G) in liver cancer. Strategy to identify miR-3144-3p and ED_miR-3144(3_A<G) target in liver cancer. **a** miR-3144-3p targets are identified with TargetScan v7.0 algorithm and their expressions were analyzed with 3 liver cancer transcriptome sequencing datasets, TCGA_LIHC, Catholic_mLIHC, and ICGC_LIRI. **b** The target candidate expression changes of miR-3144-3p in overt liver cancer, Catholic_mLIHC. **c** ED_miR-3144(3_A<G) targets are identified with TargetScan v7.0 algorithm and their expressions were analyzed with 3 liver cancer transcriptome sequencing datasets, TCGA_LIHC, Catholic_mLIHC, and ICGC_LIRI. **d** The target candidate expression changes of ED_miR-3144(3_A<G) in overt liver cancer, Catholic_mLIHC.

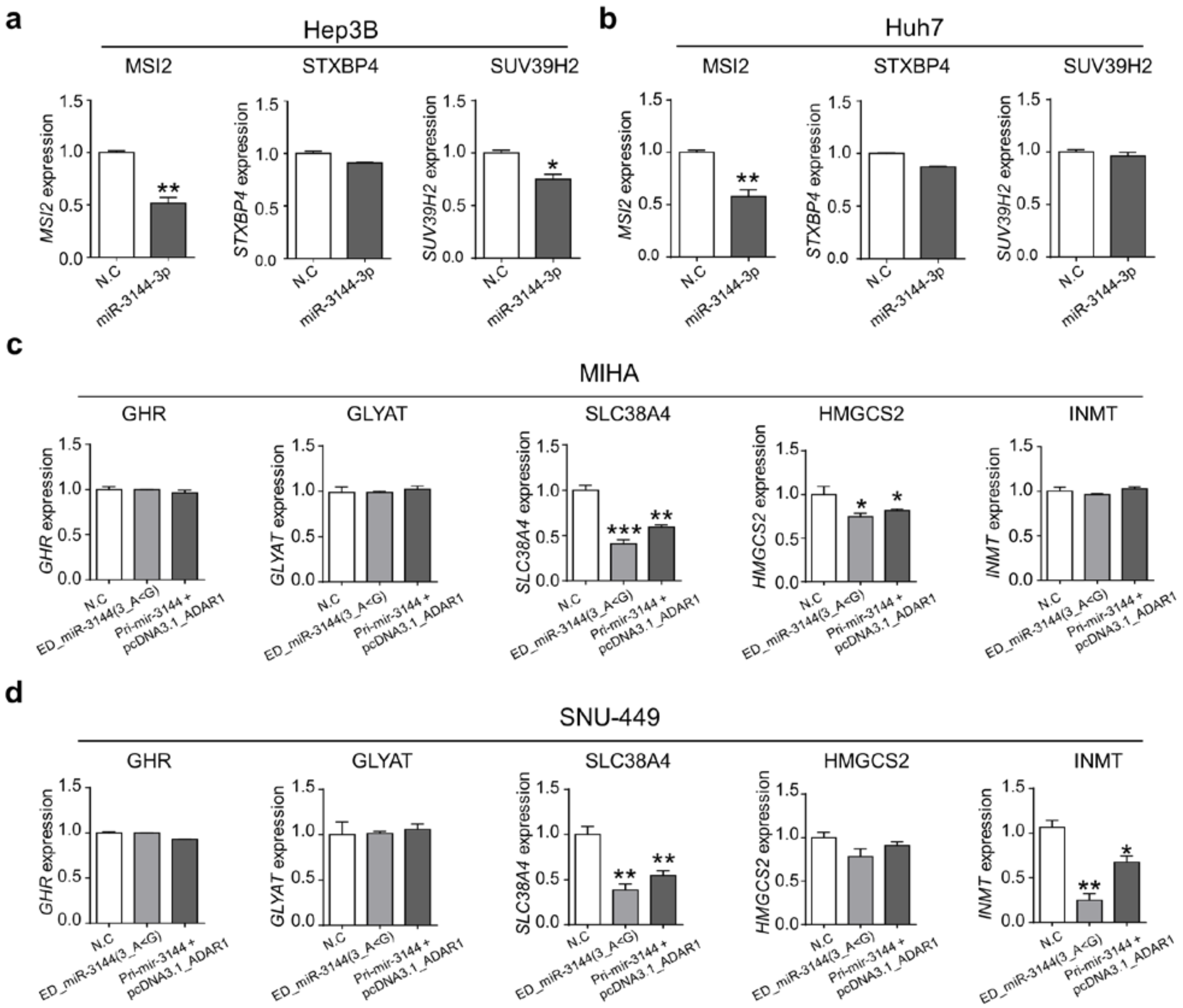

Supplementary Fig. 6. Target validation of canonical miR-3144-3p and ED_miR-3144(3_A<G). Target validation of miR-3144-3p and ED_miR-3144(3_A<G) with qRT-PCR. **a** Hep3B and **b** Huh7 were transfected with negative control (N.C) or miR-3144-3p mimics and the expression of the three target candidates was measured by qRT-PCR. The expression of the five target candidates were analyzed after transfection of ED_miR-3144(3_A<G) or pcDNA3.1_ADAR1-p110 in the background of transfected primary mir-3144 (Pri-mir-3144) in **c** MIHA and **d** SNU-449 cells with qRT-PCR. All data are shown as the mean ± SEM. *P < 0.05, **P < 0.01, ***P < 0.001 by unpaired student's t test.

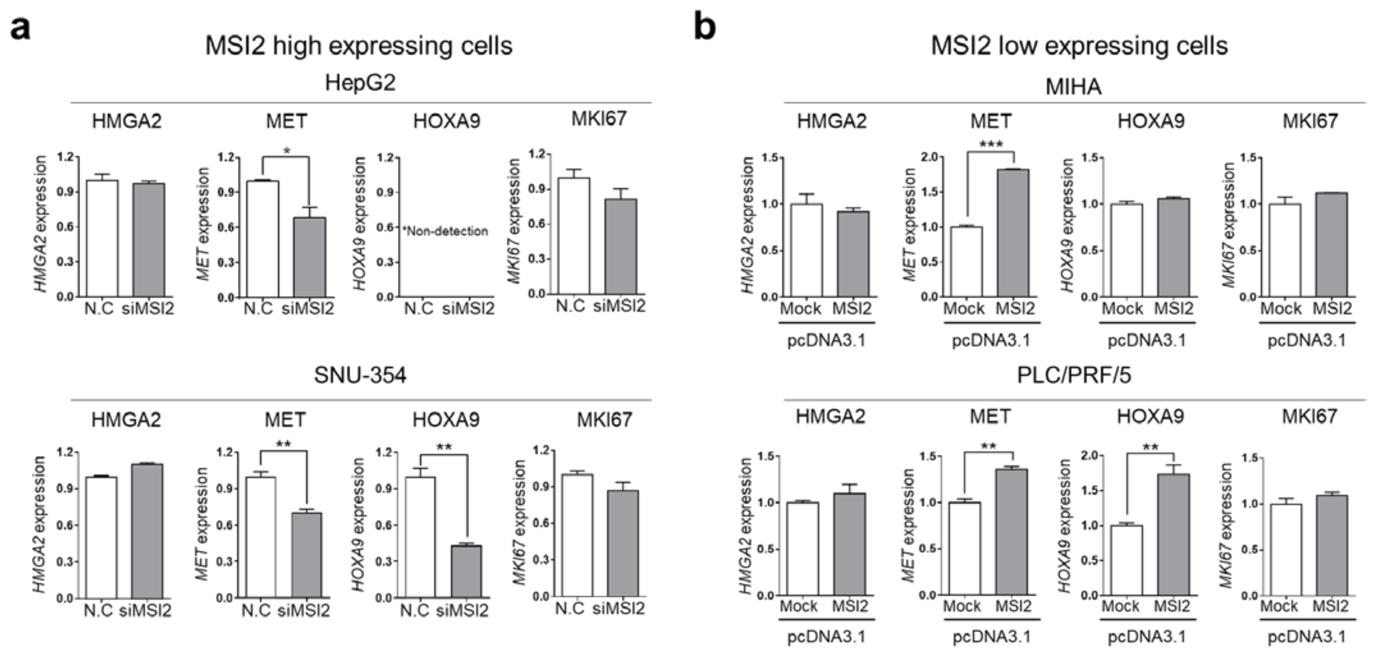

Supplementary Fig. 7. Selective regulation of MET by MSI2 in liver cancer. Expression validation of MSI2 target candidates by qRT-PCR. **a** Negative control siRNA (N.C) or siMSI2 was transfected to two liver cancer cells (HepG2 and SNU-354), and the expression was validated by qRT-PCR. **b** MIHA and PLC/PRF/5 cells were transfected with pcDNA3.1_Mock or pcDNA3.1_MSI2 and the expression of the candidate genes were measured with qRT-PCR. All data are shown as the mean ± SEM. **P* < 0.05, ***P* < 0.01, ****P* < 0.001 by unpaired student's *t* test.

a

Pearson correlation r (with ADAR1)	TCGA_LIHC	ICGC_LIRI	GSE77314	Catholic_mLIHC
MSI2	0.489 *** (n=421)	0.472 *** (n=439)	0.525 *** (n=100)	0.368 *** (n=119)
SLC38A4	-0.138 ** (n=421)	-0.233 *** (n=439)	-0.230 * (n=100)	-0.270 ** (n=119)

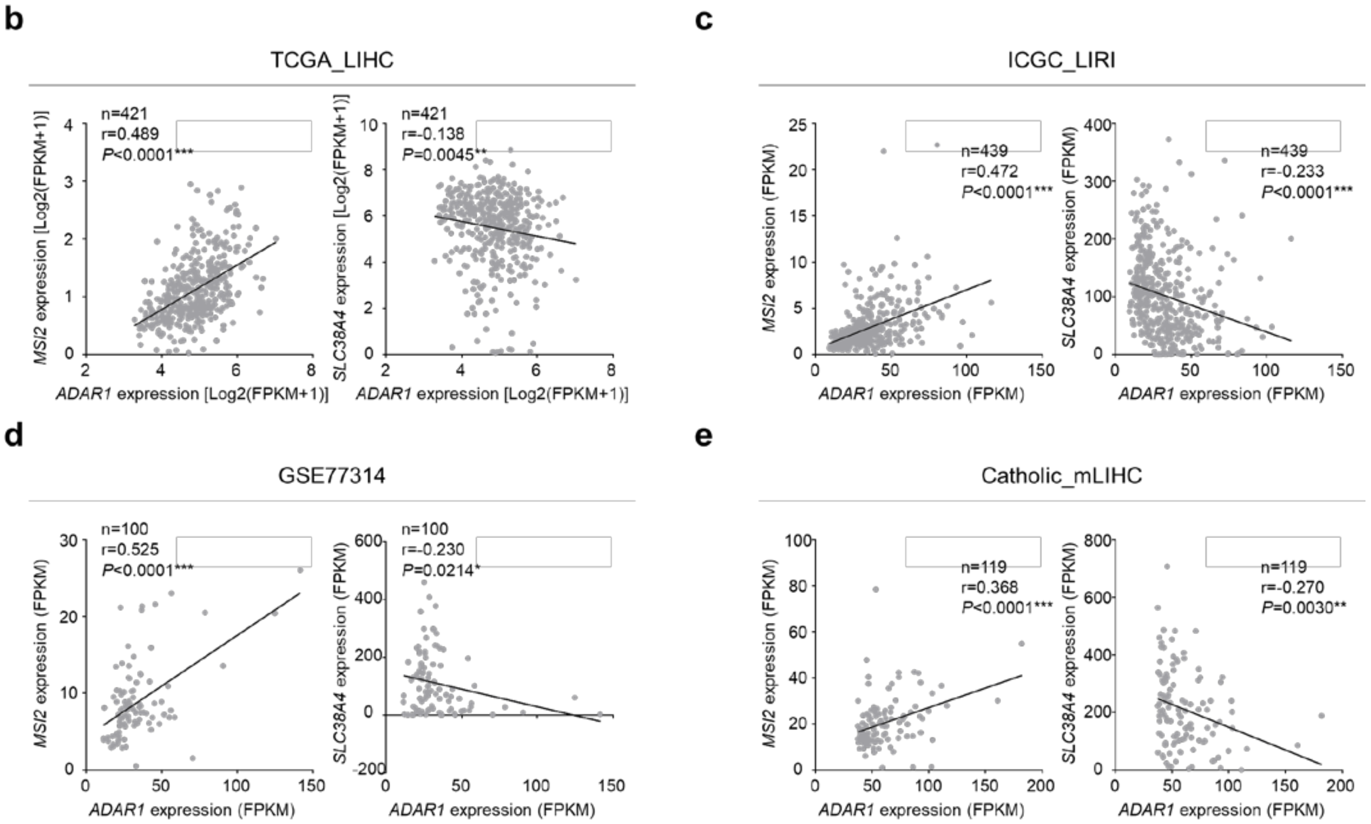

Supplementary Fig. 8. Correlation analysis of the *ADAR1* with *MSI2* and *SLC38A4* in liver cancer. a Correlation analysis of *ADAR1* mRNA level with *MSI2* and *SLC38A4* mRNA level in the transcriptomic liver cancer patient's datasets of **b** TCGA_LIHC, **c** ICGC_LIRI, **d** GSE77314, **e** Catholic_mLIHC datasets. *$P < 0.05$, **$P < 0.01$, ***$P < 0.001$ by Pearson's coefficient tests.
